# Investigating N_2_ Fixation Using a Bulky Fe(bisphosphine)_2_ Framework

**DOI:** 10.1002/chem.202502688

**Published:** 2025-11-28

**Authors:** Andrew D. Crawford, Laurence R. Doyle, Samuel J. Horsewill, William K. Myers, Daniel J. Scott, Andrew E. Ashley

**Affiliations:** ^1^ Department of Chemistry Imperial College London London UK; ^2^ Department of Chemistry University of Bath Claverton Down Bath UK; ^3^ Inorganic Chemistry Laboratory University of Oxford Oxford UK

**Keywords:** ammonia, hydrazine, iron, nitrogen fixation, phosphine ligands

## Abstract

An iron(0) dinitrogen complex incorporating highly sterically encumbered bisphosphine ligands, Fe(N_2_)(dibpe)_2_ (dibpe = *i*Bu_2_PCH_2_CH_2_P*i*Bu_2_), ***^i^* ^Bu^1·N_2_**, has been prepared and thoroughly characterized, and its N_2_ fixation reactivity assessed. ***^i^* ^Bu^1·N_2_** is a more hindered analogue of Fe(N_2_)(depe)_2_ (depe = Et_2_PCH_2_CH_2_PEt_2_), **^Et^1·N_2_**, which has previously been shown to be an efficient N_2_ reduction catalyst with unusual selectivity for N_2_H_4_, and it was anticipated that greater bulk might make ***^i^* ^Bu^1·N_2_** less prone to deleterious side reactivity, improving performance. The N_2_ ligand in ***^i^* ^Bu^1·N_2_** displays a similar degree of activation to **^Et^1·N_2_**, and the two complexes can stoichiometrically fix N_2_ with similarly high efficiency upon treatment with suitable acids, giving mixtures of NH_3_ and N_2_H_4_. However, attempts to catalytically fix N_2_ via treatment of ***^i^* ^Bu^1·N_2_** with mixtures of excess acids and reductants led to poor results. Mechanistic investigations implicate a combination of more sluggish reaction kinetics and weaker binding of N_2_ to the intermediate Fe(I) cation [Fe(dibpe)_2_]^+^, [***^i^* ^Bu^1**]^+^, whose study was aided by isolation as its BAr^F^_4_^−^ salt (Ar^F^ = 3,5‐*bis*(trifluoromethyl)phenyl), and which has also been fully characterized. This weak interaction hinders regeneration of the initial ***^i^* ^Bu^1·N_2_** via reduction of [***^i^* ^Bu^1·N_2_**]^+^, which appears to be necessary to close the catalytic cycle.

## Graphical abstract

The bulky Fe(0) N_2_ complex Fe(N_2_)(dibpe)_2_ has been prepared and efficiently produces NH_3_/N_2_H_4_ upon treatment with acid, similarly to previously investigated Fe(N_2_)(depe)_2_. However, it performs much less effectively during attempted catalytic N_2_ fixation, allowing investigation of the crucial role of steric hindrance in steps such as N_2_ binding and protonation.

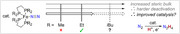

## 1 Introduction

**SCHEME 1:**
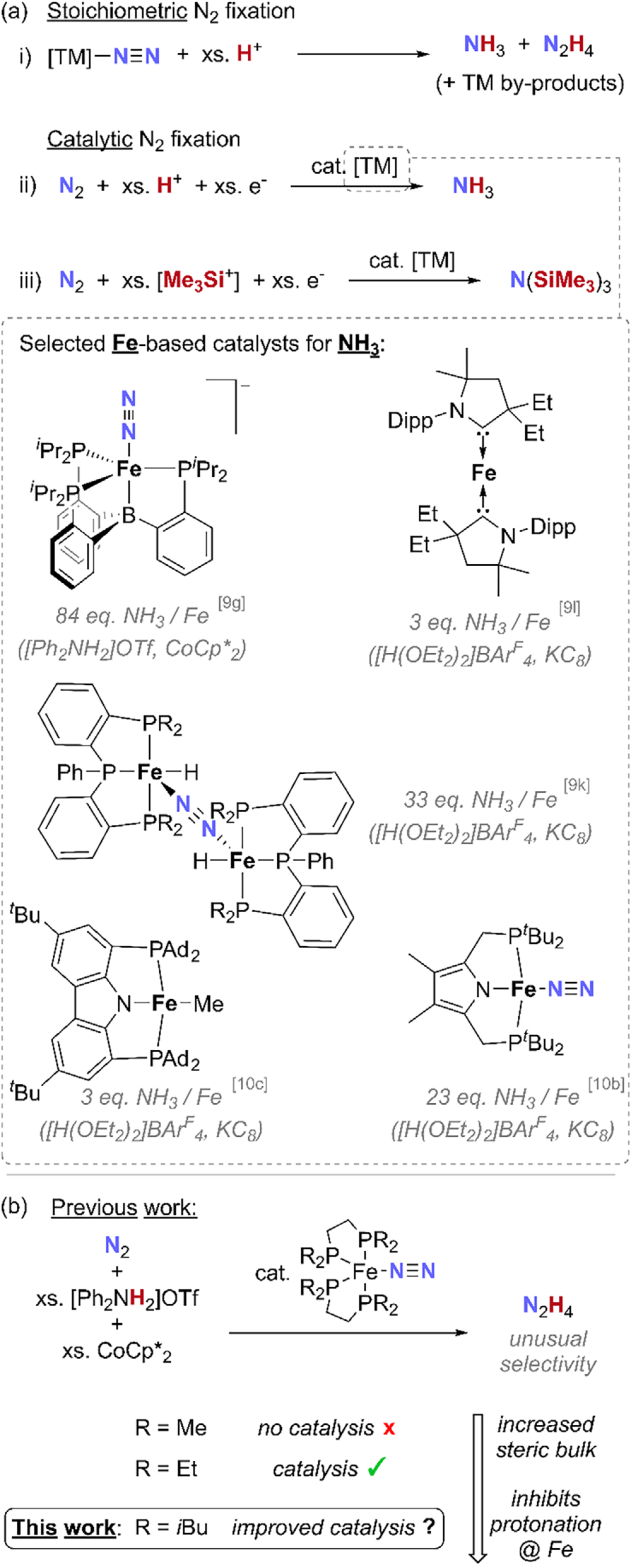
(a) General stoichiometric and catalytic fixation of N_2_ mediated by molecular TM complexes, including examples of Fe‐based catalysis, and (b) reduction of N_2_ to N_2_H_4_ catalyzed by Fe(N_2_)(PP)_2_ (PP = R_2_PCH_2_CH_2_PR_2_; [Me_3_Si^+^]. = Me_3_SiX, X = Cl/Br/I/OTf).

In recent years there has been great interest in the development of transition metal (TM) complexes capable of mediating the fixation of inert molecular N_2_ into chemically useful N‐containing compounds [1, 2, 3]. Stoichiometrically, this is typically achieved through protonation of low‐valent TM·N_2_ complexes using suitable Brønsted acids to yield the azanes NH_3_ and N_2_H_4_ (Scheme 1a, i) [4]. These reactions may sometimes be rendered catalytic by the addition of excess H^+^ and e^−^ sources under an atmosphere of N_2_ (Scheme 1a, ii) [5, 6, 7]. Analogous use of the Lewis acid Me_3_SiCl in place of Brønsted acids had also led to numerous protocols for the catalytic transformation of N_2_ into N(SiMe_3_)_3_ (Scheme 1a, iii), again in the presence of additional electron sources [8, 9, 10, 11, 12].

Much of the motivation for the study of these reactions lies in the related industrial and biological processes for N_2_ fixation, which are among the most important of all global chemical reactions. In both cases, study of the active Haber‐Bosch (industrial) [13]. or nitrogenase (biological) [14, 15] catalysts is highly challenging, and synthetic molecular catalysts can provide useful analogues that are much more amenable to mechanistic investigation. This is amplified by the ease with which molecular complexes can be modified, allowing the importance of specific structural features (e.g., steric bulk, coordination geometry) to be assessed in a controlled manner via direct comparisons between different, rationally tuned analogues.

Given the presence of Fe in both the Mittasch catalyst (used by the Haber‐Bosch process) and all known nitrogenases (FeMo/FeFe/FeV) [13, 14, 15], it is unsurprising that the use of molecular Fe complexes has attracted particular interest for the fixation of N_2_ [16]. Notable work by the groups of Peters [17, 18, 19, 20, 21, 22, 23, 24, 25, 26, 27, 28], Nishibayashi [29, 30, 31, 32, 33], and Mezailles [34], among others [35, 36, 37, 38, 39, 40, 41, 42, 43, 44, 45, 46, 47, 48, 49] has exposed a variety of Fe‐based homogeneous molecular complexes capable of catalytically transforming N_2_ to NH_3_. Among our own contributions to this field, [50, 51, 52, 53, 54] we have shown that a surprisingly simple bisphosphine complex, Fe(N_2_)(depe)_2_ (depe = Et_2_PCH_2_CH_2_PEt_2_; **^Et^1·N_2_**), is capable of efficient stoichiometric N_2_ fixation to give NH_3_ and N_2_H_4_ (up to 55% electron yield) [54]. **^Et^1·N_2_** was subsequently shown to be effective in the catalytic fixation of N_2_ (up to 25 N_2_ reduced per Fe) [53]. Notably, this was found to proceed with unprecedented selectivity for N_2_H_4_, which was formed almost exclusively, instead of the more commonly observed NH_3_ (Scheme 1b) [18, 19].

**SCHEME 2:**
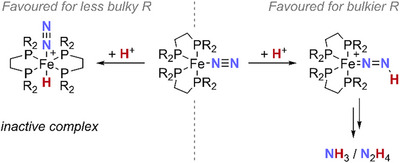
Competing protonation of complexes **1·N_2_** at Fe (left) or N_β_ (right), leading to complex deactivation or N_2_ functionalization, respectively.

Interestingly, replacing **^Et^1·N_2_** with the slightly smaller but otherwise very similar complex Fe(N_2_)(dmpe)_2_ (dmpe = Me_2_PCH_2_CH_2_PMe_2_; **^Me^1·N_2_**) led to inferior results in analogous reactions (see refs. 53 and 54 and Table S2, entries 16 and 17). This was proposed to be due to rapid, thermodynamically favored protonation of **^Me^1·N_2_** at the metal, leading to oxidized Fe(II) hydrides that are inactive toward N_2_ fixation [54]. In comparison, the bulkier depe ligands in **^Et^1·N_2_** should confer greater steric protection, thereby channeling reactivity away from this pathway and toward productive protonation at the more accessible terminal N_β_, which is kinetically favored (Scheme 2).

Extrapolation from this conclusion suggests that further increases in the steric bulk of the bisphosphine ligand might continue to lead to improved catalytic performance. To test this hypothesis and to provide further insight into the mechanistic factors that underpin the catalytic N_2_ reduction activity of **^Et^1·N_2_**, we report herein the preparation of the analogous complex Fe(N_2_)(dibpe)_2_ (dibpe = *i*Bu_2_PCH_2_CH_2_P*i*Bu_2_; ***^i^* ^Bu^1·N_2_**) and assess its performance in stoichiometric and catalytic N_2_ fixation. Consistent with expectations, ***^i^* ^Bu^1·N_2_** is an effective mediator of the former, but in contrast with expectations, it fails to achieve the latter. Accompanying mechanistic studies help to account for this surprising apparent contradiction.

## 2 Results and Discussion

### 2.1 Synthesis and Characterization of *^i^* ^Bu^1·N_2_

**SCHEME 3:**
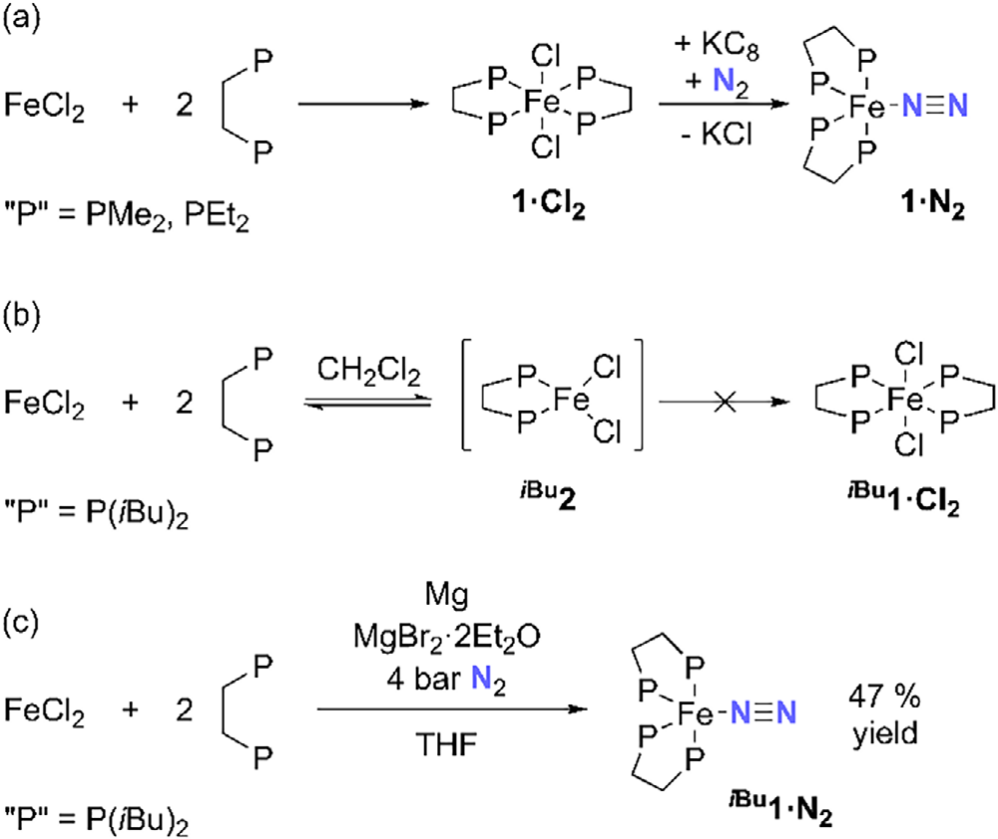
(a) Previous synthesis of **^Me^1·N_2_** and **^Et^1·N_2_**; [4, 54, 55, 56, 57] (b) attempted synthesis of ***^i^* ^Bu^1·Cl_2_**; and (c) synthesis of ***^i^* ^Bu^1·N_2_**.

**FIGURE 1:**
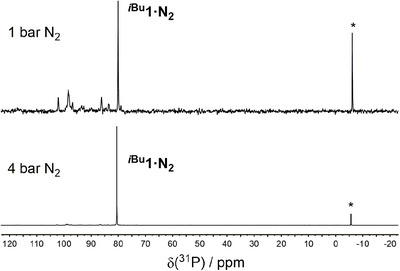
^31^P{^1^H} NMR spectra for the synthesis of ***^i^* ^Bu^1·N_2_** under 1 bar (top) or 4 bar (bottom) N_2_ pressure, at equal concentrations (* = PPh_3_ in capillary insert). Spectra not to equal scale (equivalent capillary insert used for both spectra).

The previously studied complexes **^Me^1·N_2_** and **^Et^1·N_2_** could both be prepared through preparation of the corresponding Fe(II) chloride precursor, FeCl_2_(PP)_2_ (PP = dmpe, **^Me^1·Cl_2_**; depe, **^Et^1·Cl_2_**), and subsequent reduction (Scheme 3a) [4, 54, 55, 56, 57]. However, attempts to isolate the dibpe‐substituted analogue ***^i^* ^Bu^1·Cl_2_** through analogous reactions were unsuccessful. Instead, the combination of FeCl_2_ and dibpe led to broad resonances at chemical shifts corresponding to the free ligand in the ^31^P{^1^H} and ^1^H NMR spectra, and solvent peaks were shifted relative to an internal standard, indicating the formation of paramagnetic species [58]. Nor was any color change observed (*cf*. vivid green colors for **^Me^1·Cl_2_** and **^Et^1·Cl_2_**) [56]. These observations are tentatively attributed to reversible formation of the high‐spin, four‐coordinate complex FeCl_2_(dibpe), ***^i^* ^Bu^2**, with coordination of a second dibpe ligand being prevented on steric grounds (Scheme 3b). Nevertheless, it was anticipated that, while the intended Fe(dibpe)_2_ core may be too bulky to accommodate the two chloride ligands in ***^i^* ^Bu^1·Cl_2_**, the formation of ***^i^* ^Bu^1·N_2_** should still be possible due to both the reduced steric profile of the N_2_ ligand and the greater size of Fe(0) relative to Fe(II). Thus, a ‘one‐pot’ synthesis of ***^i^* ^Bu^1·N_2_** from FeCl_2_, dibpe, and reductant under an N_2_ atmosphere was pursued, in the hope that in situ reduction of the postulated ***^i^* ^Bu^2** would allow a second dibpe ligand to coordinate after loss of chloride. Gratifyingly, the reaction of FeCl_2_ and dibpe with a combination of Mg and MgBr_2_·(OEt_2_)_2_ in THF under N_2_ led to the formation of an orange/red solution similar in color to **^Me/Et^1·N_2_** (and clearly distinct from colorless ***^i^* ^Bu^2** or green **^Me^1·Cl_2_/ ^Et^1·Cl_2_**), and extraction into pentane and crystallization at low temperature conveniently afforded the target complex ***^i^* ^Bu^1·N_2_** in reasonable yield (47%; Scheme 3c). Notably, the use of an elevated N_2_ pressure (4 bar) was crucial to achieve a useful yield in this reaction. In its absence, while ***^i^* ^Bu^1·N_2_** was still formed, numerous side products were also evident by ^31^P NMR spectroscopy (Figure 1). Associated small peaks in the hydride region of the ^1^H NMR spectrum were also observed, and it is proposed that these may arise from cyclometallation of the *i*Bu groups of the dibpe ligands, as has been reported for related reduced Fe(PP)_2_ species [59]. This side‐reactivity could be facilitated by slower binding of N_2_ to the bulky Fe center (*vide infra*).

Compound ***^i^* ^Bu^1·N_2_** is stable for at least several months when stored at −30°C under Ar or N_2_ in the solid state and is also stable for extended periods in aliphatic hydrocarbon solutions. However, rapid decomposition was observed in aromatic solvents such as benzene, and slower decomposition was also observed in donor solvents, which is believed to be due to displacement by solvent of the N_2_ ligand and subsequent cyclometallation of the dibpe ligand (as seen for N_2_‐free Fe^0^(depe)_2_) [60, 61] over the course of several hours. As a result, where THF or Et_2_O have been employed as solvents for subsequent studies, these solutions were prepared immediately prior to use.

**FIGURE 2:**
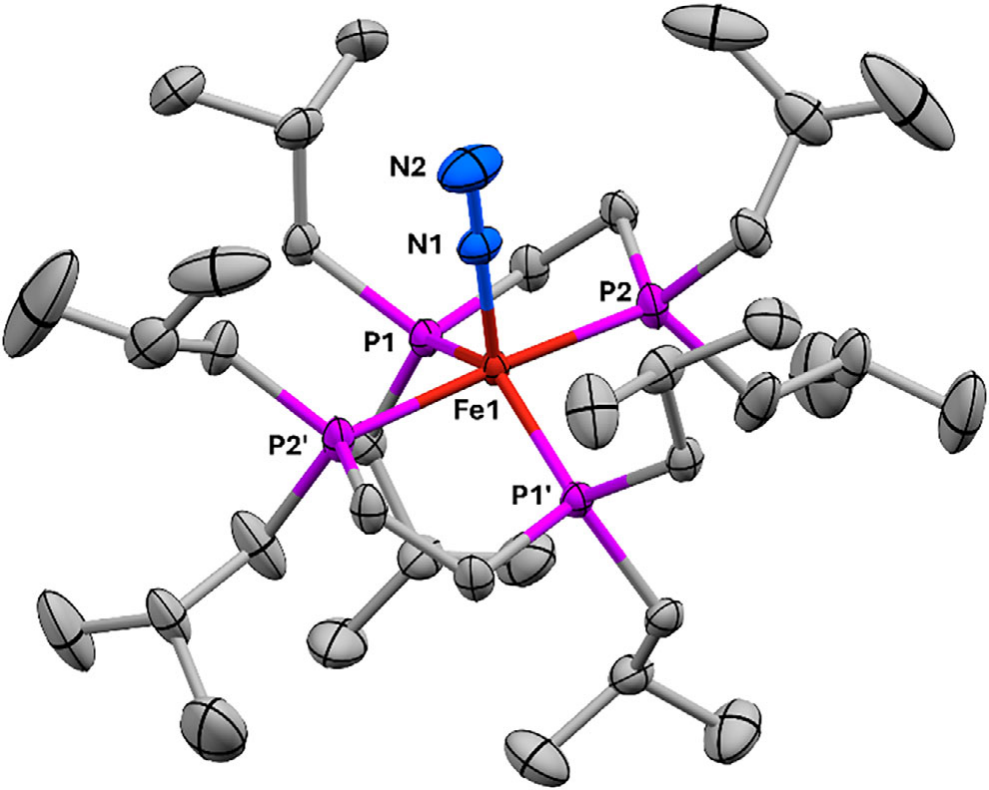
X‐ray crystallographic structure of ***^i^* ^Bu^1·N_2_**. Selected bond lengths: Fe1‐P1, 2.1997(5); Fe1‐P2, 2.2077(5); Fe1‐N1, 1.791(3); N1‐N2, 1.125(4) Å. Selected bond angles: P1‐Fe1‐P1′, 140.72(3)°; P2‐Fe1‐P2′, 176.61(4)°; Fe1‐N1‐N2, 180.0° (required by symmetry). Ellipsoids drawn at 50% probability. Minor occupancy disorder positions and H atoms omitted for clarity. Fe atoms are shown in rust, P in magenta, C in grey, and N in blue. The two ligands are related by C_2_ symmetry about the Fe‐N_2_ axis, and one of the *i*Bu groups is disordered over two independent orientations, only one of which is shown.

Complex ***^i^* ^Bu^1·N_2_** has been fully characterized and shows a singlet ^31^P NMR resonance at 79.7 ppm in *per*‐deuterated methylcyclohexane. Single crystals suitable for analysis by X‐ray diffraction analysis (XRD) were grown from pentane at −30°C and the solid‐state structure confirms the proposed formulation, showing a geometry similar to both **^Et^1·N_2_** and **(^Me^1)_2_·N_2_** (the latter being a dimeric structure with a bridging N_2_ ligand that exists in equilibrium with monomeric **^Me^1·N_2_**) [54]. Nevertheless, ***^i^* ^Bu^1·N_2_** shows a significant distortion away from the expected trigonal bipyramidal geometry and toward a square‐based pyramidal structure (Figure 2). This can be attributed to the increased steric demands of the dibpe ligand and is quantified by a τ parameter of 0.6 (τ = 0 for idealized square‐based pyramidal, 1 for ideal trigonal bipyramidal geometries; [62] *cf*. τ = 0.89 for **^Et^1·N_2_** [63], τ = 0.99, 1.05 for **(^Me^1)_2_·N_2_** [54]).

The crystallographic N—N distance of 1.125(4) Å in ***^i^* ^Bu^1·N_2_** is identical (within error) to that of **^Et^1·N_2_** (1.142(7) Å) [63]. However, this is known to be a poor measure of the degree of N_2_ activation in such complexes [16]. To obtain a more reliable indication of this key parameter, solution‐phase IR spectroscopy was carried out in Et_2_O, which revealed an absorption at 1984 cm^−1^ that was assigned to the N—N stretching frequency. This assignment was confirmed through preparation of the isotopically labelled complex ***^i^* ^Bu^1·^15^N_2_**, for which this feature was shifted to a higher frequency of 1899 cm^−1^ (ν(^14^N_2_)/ν(^15^N_2_) = 1.045 (experimental), 1.035 (theoretical; simple harmonic oscillator model)). The observed frequency is slightly higher than those for related Fe(N_2_)(PP)_2_ complexes (e.g. 1955 cm^−1^ for **^Et^1·N_2_**, 1975 cm^−1^ for **^Me^1·N_2_**) [56, 57] and is consistent with a slightly less activated N≡N bond [64]. This conclusion is further supported by ^15^N NMR spectroscopic studies of ***^i^* ^Bu^1·^15^N_2_**, with the ^15^N{^1^H} spectrum showing two doublets at very close chemical shifts (δ = 331.1, 330.8 ppm, Δδ  = 0.3 ppm), indicating that the N atoms are in very similar chemical environments. For comparison, **^Me^1·N_2_** (for example) shows a larger separation of Δδ = 1.8 ppm, although the coupling constants ^1^ *J*_NN_ for the two compounds are similar (6.5 Hz and 5.9 Hz, respectively) [54].

### 2.2 N_2_ Reduction Using *^i^* ^Bu^1·N_2_

With compound ***^i^* ^Bu^1·N_2_** in hand, investigations were carried out into its capacity for N_2_ fixation, beginning with stoichiometric N_2_ reduction (i.e. acidification with no external reductant, *cf*. Scheme 1a, i). Previously, excellent results had been obtained by treating **^Et^1·N_2_** with the strong acid TfOH in pentane at ambient temperature (20°C) [54] Gratifyingly, very similar results could be obtained using ***^i^* ^Bu^1·N_2_**, with mixtures of NH_3_ and N_2_H_4_ being obtainable with electron yields up to 51% (*cf*. up to 55% using **^Et^1·N_2_**; for full details, including discussion of minor differences between the two systems, see SI Section 2.1 and Table S2). The fate of the final Fe/dibpe fragments in these reactions was not investigated in detail but is likely to include protonated bisphosphine ([dipbe(H)_2_]^2+^) and Fe^2+^ salts, in line with previous studies of **^Et^1·N_2_** [53].

**SCHEME 4:**
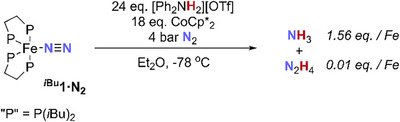
Attempted catalytic fixation of N_2_ by ***^i^* ^Bu^1·N_2_** in the presence of CoCp*_2_. All other reaction conditions tested gave inferior results (see Table S3).

Having established that stoichiometric N_2_ fixation can be achieved with high efficiency using ***^i^* ^Bu^1·N_2_**, investigations proceeded to the use of ***^i^* ^Bu^1·N_2_** for the catalytic reduction of N_2_. For these studies, CoCp*_2_ (Cp* = C_5_Me_5_) was used as an additional chemical reductant, having been shown to give optimal performance in analogous reactions using **^Et^1·N_2_** [53]. Surprisingly, however, with ***^i^* ^Bu^1·N_2_** these reactions were found to give significantly inferior results. Indeed, even after surveying a range of different reaction conditions (see Table S3), the best result using ***^i^* ^Bu^1·N_2_** yielded only 1.6 eq. of fixed nitrogen atoms (Scheme 4), which is an order of magnitude lower than for **^Et^1·N_2_** (up to *ca*. 30 eq.) and falls below the threshold of formal catalysis. Moreover, only trace N_2_H_4_ was observed (*vide infra*).

### 2.3 Synthesis, Characterization, and Mechanistic Role of [*^i^* ^Bu^1]^+^

Though unexpected, the poor performance of ***^i^* ^Bu^1·N_2_** under the catalytic reaction conditions may partially be explained by the low reaction temperatures, which are needed to minimize the rate of unproductive H_2_ evolution (via direct side reaction between the acid and reductant). This was found to lead to significantly diminished results for the stoichiometric acidification procedure (see SI Section 2.1 and Table S2), likely due to kinetic factors. This suggests that the poor catalytic performance of ***^i^* ^Bu^1·N_2_** is at least partially due to slow N_2_ protonation and reduction kinetics (due in turn to the bulky dibpe ligands), rendering these steps uncompetitive with the competing, uncatalyzed H_2_ evolution. Nevertheless, the magnitude of the apparent discrepancy in performance between the two regimes (and between ***^i^* ^Bu^1·N_2_** and **^Et^1·N_2_**) suggests that other factors could also be relevant, which prompted further mechanistic investigations. In particular, it was speculated that the contrast between the excellent performance of the optimized stoichiometric reaction and the poor performance of the optimized catalytic reaction may imply difficulties in regenerating the initial ***^i^* ^Bu^1·N_2_**, as required to close the catalytic cycle, rather than just the transformation of ***^i^* ^Bu^1·N_2_** into NH_3_/N_2_H_4_.

**SCHEME 5:**
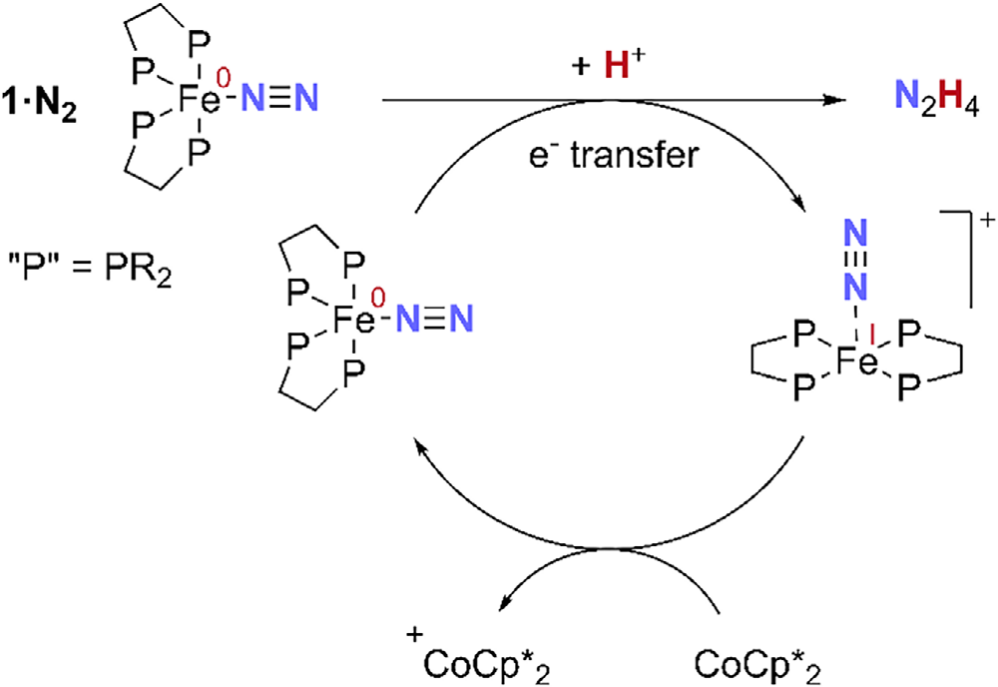
A simplified, outline mechanism for N_2_ fixation mediated by complexes **1·N_2_**.

Our previous studies of **^Et^1·N_2_** have highlighted the importance of the Fe(0/I) redox couple during N_2_ fixation [53, 54]. During stoichiometric acidification this strongly reducing couple is believed to be crucial in supplying electrons for the reduction of N_2_ to N*_x_* H*_y_*. Indeed, it is notable that the electron yields achieved for stoichiometric acidification of **1·N_2_** seem to be limited to a maximum of approx. 50%. Since calculation of these yields assumes oxidation of Fe(0) to Fe(II) [4, 49] this may imply that, in practice, only one electron per Fe is readily available for N_2_ reduction, presumably originating from the most reducing couple present, that is, Fe(0/I) only. Catalytic turnover can then be achieved by re‐reduction of the resulting Fe(I) species by an external reductant, to regenerate the Fe(0) starting material (Scheme 5) [65, 66].

**FIGURE 3:**
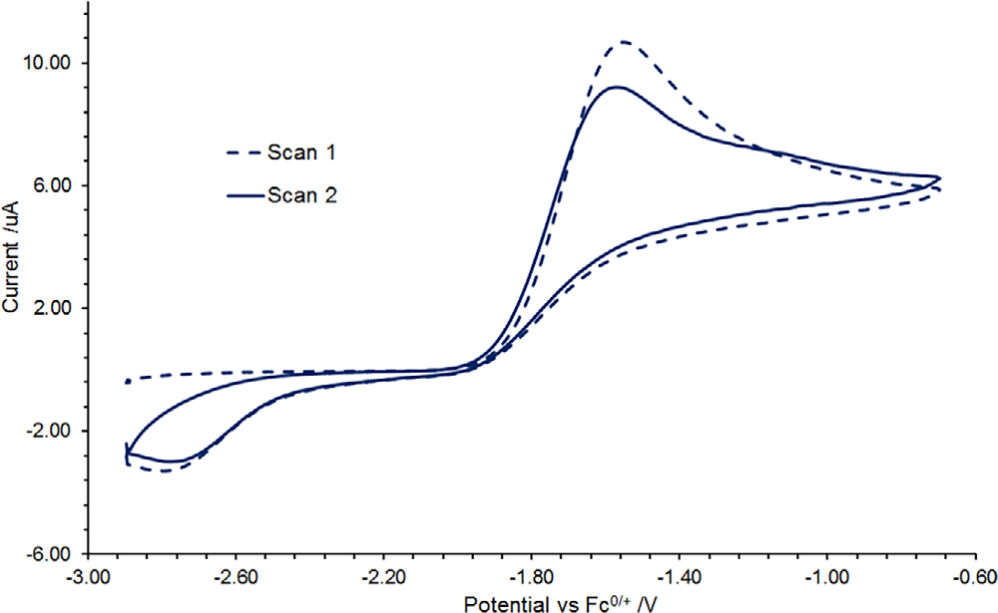
Cyclic voltammogram of ***^i^* ^Bu^1·N_2_** in Et_2_O under N_2_ using [*n*Bu_4_N][BAr^F^_4_] electrolyte, referenced vs. Fc/Fc^+^ (200 mV/s).

To assess the reducing power of the Fe(0/I) couple in ***^i^* ^Bu^1·N_2_**, electrochemical investigations were carried out. Cyclic voltammetry (CV) measurements on an Et_2_O solution of ***^i^* ^Bu^1·N_2_** under an N_2_ atmosphere ([*n*Bu_4_N][BAr^F^_4_] supporting electrolyte, Ar^F^  = 3,5‐*bis*(trifluoromethyl)phenyl) revealed that initial oxidation of ***^i^* ^Bu^1·N_2_** occurs irreversibly at very low potential (*E*_p,ox_ ∼−1.56 V vs Fc/Fc^+^ at 200 mV/s, Fc = ferrocene; Figure 3). While this irreversibility prevents a simple evaluation of the precise Fe(0/I) redox potential, it is clear that ***^i^* ^Bu^1·N_2_** is a powerful reductant in line with other, similar Fe(N_2_)(PP)_2_ species, albeit not quite as powerful as **^Et^1·N_2_** (for which the equivalent redox potential is measured as −2.0 V vs. Fc/Fc^+^, with E_p,ox_ ∼ −1.88 V) [53, 54]. Notably, however, **^Et^1·N_2_** oxidation was fully reversible over scan rates between 100 and 2000 mV/s. The contrasting irreversibility for ***^i^* ^Bu^1·N_2_** is consistent with rapid loss of the N_2_ ligand upon oxidation and hence with a weaker interaction of N_2_ with the oxidized [Fe(dibpe)_2_]^+^ core vs. [Fe(depe)_2_]^+^. This helps to explain the need for elevated N_2_ pressures during the synthesis of ***^i^* ^Bu^1·N_2_** (*vide supra*), since the Fe(0) product of this reaction is presumably formed via Mg reduction of the Fe(I) intermediate.

**SCHEME 6:**
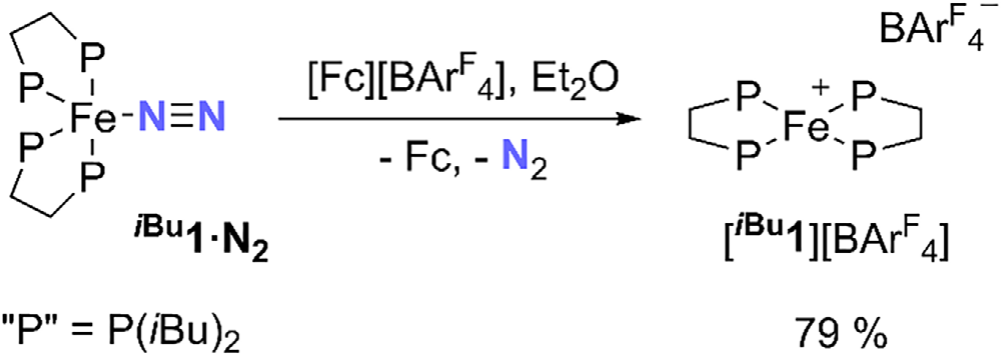
Synthesis of [***^i^* ^Bu^1**][BAr^F^_4_]. Fc = Fe(C_5_H_5_)_2_.

**FIGURE 4:**
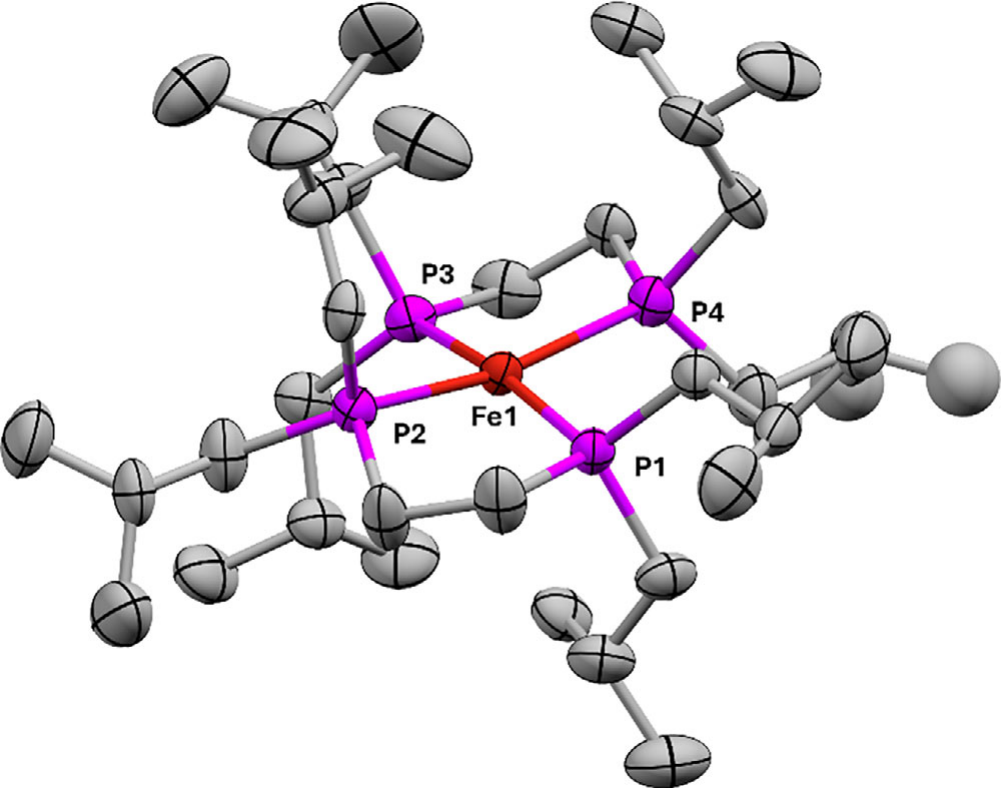
X‐ray crystallographic structure of [***^i^* ^Bu^1**][BAr^F^_4_]. Selected bond lengths: Fe1‐P1, 2.2449(7); Fe1‐P2, 2.2390(8); Fe1‐P3, 2.2433(7); Fe1‐P4, 2.281(7); Fe1‐P4', 2.245(9) Å. Selected bond angles: P1‐Fe1‐P3, 171.81(3)°; P2‐Fe1‐P4, 166.86(15)°; P2‐Fe1‐P4′, 173.9(3)° (P4′ not pictured). Ellipsoids drawn at 50% probability. Minor occupancy disorder positions in dibpe ligands, H atoms, and [BAr^F^_4_]^−^ anion omitted for clarity. Fe atoms are shown in rust, P in magenta, and C in grey. P9 is disordered over two possible positions, resulting in two orientations of the dibpe ligand. Only the major occupancy orientation of that ligand is shown.

In line with this interpretation of the CV results, treatment of an ethereal solution of ***^i^* ^Bu^1·N_2_** with [Fc][BAr^F^_4_] led to an immediate color change to deep purple. Notably, a similar transient purple color had also been observed during many of the earlier ***^i^* ^Bu^1·N_2_** acidification reactions. Removal of solvent *in vacuo*, washing with pentane, and recrystallization from Et_2_O yielded the four‐coordinate, 15 valence‐electron Fe(I) product [Fe(dibpe)_2_][BAr^F^_4_] ([***^i^* ^Bu^1**][BAr^F^_4_]; Scheme 6). XRD analysis of single crystals of [***^i^* ^Bu^1**][BAr^F^_4_] grown from Et_2_O at −30°C revealed a square‐planar coordination geometry with only a slight tetrahedral distortion (Figure 4), very similar to the related depe‐substituted salt [**^Et^1**][BAr^F^_4_] [51]. Notably, the solid‐state structure showed no evidence of an N_2_ ligand bound to the Fe center, even under N_2_ atmosphere (*vide infra*).

Analysis by the Evans NMR method [67] provided a solution‐phase magnetic moment of 1.98 µ_B_, consistent with a low‐spin S  =  ½ electronic configuration (no observable change in magnetic moment is evident between 25 and −80°C, indicating that deviation from the spin‐only value of 1.73 µ_B_ is due to an orbital contribution to µ_eff_ rather than thermal population of a higher spin state). As such, solutions of [***^i^* ^Bu^1**][BAr^F^_4_] are silent to ^31^P NMR spectroscopy, although a pair of characteristic broad resonances were visible in the room temperature ^1^H NMR spectrum (approx. −1.3 and −12.6 ppm in Et_2_O; similar signals were observed for [**^Et^1**][BAr^F^_4_]) [51]. UV‐vis spectroscopy, meanwhile, revealed that the purple color of [***^i^* ^Bu^1**][BAr^F^_4_] is due to a number of electronic absorptions in the visible region, most notably a strong absorption at 520 nm (2960 M^−1^ cm^−1^).

**FIGURE 5:**
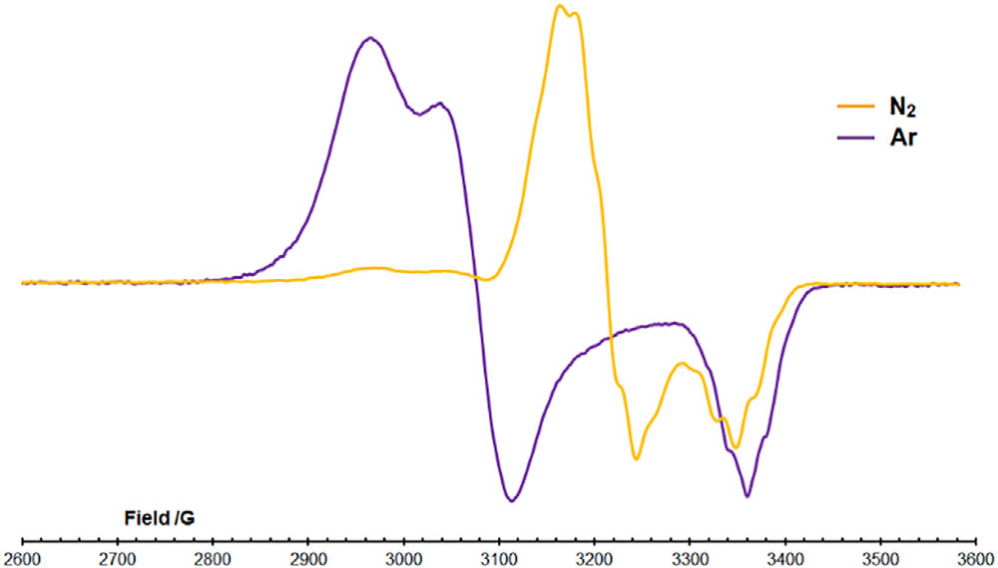
CW X‐band EPR spectra of [*^i^* **^Bu^1**][BAr^F^_4_] recorded at 40 K as toluene glasses. The signal under Ar is shown in purple; degassing the tube and backfilling with N_2_ results in the yellow signal.

Upon cooling below approx. 40°C under an atmosphere of N_2_, solutions of [***^i^* ^Bu^1**][BAr^F^_4_] were observed to lose their purple coloration. Correspondingly, the UV‐vis features associated with [***^i^* ^Bu^1**][BAr^F^_4_] were no longer observed; instead, a new feature was observed at 365 nm. Extensive studies of the depe analogue [**^Et^1**][BAr^F^_4_] have shown an analogous color change to be due to reversible binding of N_2_ to the [Fe(PP)_2_]^+^ core, although in that case the transition was observed at significantly higher temperature [51]. Furthermore, while EPR spectroscopic analysis (CW, X‐band) of [***^i^* ^Bu^1**][BAr^F^_4_] under Ar at 40 K showed a clearly rhombic signal (consistent with an S  =  ½ species, g = [2.2697, 2.1798, 1.9973], g_av_ = 2.15), under N_2_ a less anisotropic signal was observed (g = [2.0038, 2.088, 2.123], g_av_  =  2.072; Figure 5 and Figure S8). These spectra are again very similar to those observed for uncoordinated [**^Et^1**][BAr^F^_4_] and N_2_‐bound [**^Et^1·N_2_**][BAr^F^_4_], respectively [51]. Collectively, these data strongly indicate reversible binding of N_2_ to the [***^i^* ^Bu^1**]^+^ cation and that this binding is favored only at low temperatures.

**FIGURE 6:**
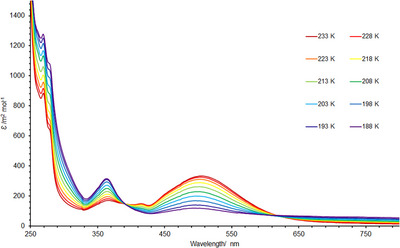
VT UV‐vis spectra of [*^i^* **^Bu^1**][BAr^F^_4_] recorded in THF (0.15 mM) under N_2_ atmosphere.

Variable‐temperature (VT) UV‐vis spectroscopy allowed the strength of this binding to be quantified (Figure 6). Van't Hoff analysis between 188 and 233 K yielded values of ΔH = −6.9(5) kcal mol^−1^ and ΔS = −25(2) cal K^−1^ mol^−1^ for N_2_ binding to [***^i^* ^Bu^1**][BAr^F^_4_] (assessed based on the intense absorption at 520 nm observed for [***^i^* ^Bu^1**][BAr^F^_4_] and literature data for the solubility of N_2_; for full details see SI Section 3). While the entropy value is similar to that obtained previously for [**^Et^1**][BAr^F^_4_] (ΔS = −27.6(1) cal K^−1^ mol^−1^), the enthalpy is significantly lower (*cf*. ΔH = −13.1(1) kcal mol^−1^ for [**^Et^1**][BAr^F^_4_]) [51]. Notably, the VT UV‐vis data indicate that N_2_ binding is incomplete even at the low temperatures investigated for catalysis (*ca*. 195 K). This confirmation that N_2_ interacts much more weakly with the bulkier [***^i^* ^Bu^1**]^+^ core suggests a further explanation for the poor catalytic performance of ***^i^* ^Bu^1·N_2_**. In the absence of strong (and rapid) binding to N_2_, it seems likely that reduction of [***^i^* ^Bu^1**]^+^ does not lead cleanly to reformation of ***^i^* ^Bu^1·N_2_**, but instead also produces significant quantities of four‐coordinate Fe^0^(dibpe)_2_ (***^i^* ^Bu^1**), which can then rapidly decompose via ligand cyclometallation reactions [59, 60, 61]. Indeed, attempted reduction of [***^i^* ^Bu^1**]^+^ with a single equivalent of CoCp*_2_ under one atmosphere of N_2_ was found to regenerate ***^i^* ^Bu^1·N_2_** alongside significant quantities of side products spectroscopically identical to those previously observed during attempts to synthesize ***^i^* ^Bu^1·N_2_** under only 1 bar of N_2_ (*cf*. Figure 2).

## 3 Conclusions

We have synthesized and thoroughly characterized the low‐valent iron dinitrogen complex Fe(N_2_)(dibpe)_2_, ***^i^* ^Bu^1·N_2_**, which is a bulkier analogue of the related complex Fe(N_2_)(depe)_2_, **^Et^1·N_2_**. This has revealed that, despite showing comparable efficiency in the stoichiometric fixation of N_2_, ***^i^* ^Bu^1·N_2_** performs much more poorly than **^Et^1·N_2_** in catalytic protocols, which mechanistic investigations suggest may be due to a combination of slower N_2_ functionalization kinetics and weaker N_2_ binding to the Fe(dibpe)_2_ moiety, particularly in the Fe(I) oxidation state. These results shed further light on the catalytic efficiency of Fe(N_2_)(depe)_2_ and suggest that it exists in a steric ‘sweet spot’: bulky enough to minimize the rate of unproductive protonation at Fe, but not bulky enough to slow N_2_ binding and subsequent protonation at N_β_ to the point that they can be outcompeted by other unproductive side‐reactions (such as H_2_ evolution and cyclometallation).

Also conspicuous is the very different distribution of azane products observed during (attempted) catalytic runs with ***^i^* ^Bu^1·N_2_** when compared with **^Et^1·N_2_** or **^Me^1·N_2_**. This is again likely due to the greater steric profile of the dibpe ligands and may specifically be attributable to the greater steric protection afforded to the proximal N_α_ site. This should hinder functionalization at this less accessible position and so disfavor the “alternating” N_2_ functionalization pathways that could lead to N_2_H_4_ formation and instead encourage “distal” pathways that form exclusively NH_3_. This would be particularly true if N_2_ reduction is achieved via H atom transfer from bulky donors such as [Cp*Co(C_5_Me_5_H)^+^], whose *in situ* formation by protonation of CoCp_2_* has been described in detail by the Peters group [23, 68].

These observations highlight the subtle and complex steric balances that underpin N_2_ reduction mediated by Fe(N_2_)(PP)_2_ complexes and the importance of comprehensive mechanistic understanding for the design of efficient catalytic systems. Similar conclusions are likely to apply to many other examples of homogeneous N_2_ reduction catalysis.

## 4 Experimental Section

### 4.1 General Methods

All chemical manipulations were performed under an N_2_ atmosphere using standard Schlenk line techniques or an MBraun Labmaster DP glovebox, unless stated otherwise. Solvents were purchased from VWR: MeOH was dried over several batches of 3 Å molecular sieves; benzene was dried over several batches of 5 Å molecular sieves; pentane and toluene were dried using an Innovative Technology Pure Solv SPS‐400; THF and Et_2_O were distilled from dark green Na/fluorenone indicator. Solvents were degassed by thorough sparging with N_2_ gas and stored in gas‐tight ampoules; pentane, benzene, toluene, and Et_2_O were stored over a K mirror. Solvents for use under Ar were thoroughly sparged with Ar (BOC, Zero grade), followed by freeze‐pump‐thaw degassing. Deuterated solvents were freeze‐pump‐thaw degassed, dried, and stored in gas‐tight ampoules over a K mirror (C_6_D_6_, methylcyclohexane‐d_14_) or 4 Å molecular sieves (THF‐d_8_, CDCl_3_). ^15^N_2_ (Cambridge Isotope Laboratories, 98% ^15^N) was transferred from a breakseal flask using a Toepler pump. All glassware and stainless steel cannulae were dried in an oven at 160°C overnight before use.

NMR spectra were recorded using Bruker AV‐400 (400.4 MHz) spectrometers. Chemical shifts, δ, are reported in parts per million (ppm). ^1^H chemical shifts are given relative to Me_4_Si and referenced internally to the residual proton shift of the deuterated solvent employed. ^19^F, ^31^P, and ^15^N chemical shifts were referenced (δ = 0) externally to CFCl_3_, 85% H_3_PO_4_ (aq), and neat CH_3_NO_2_, respectively. ^1^H and ^31^P NMR spectra of solutions prepared in nondeuterated solvents incorporate an internal reference capillary containing a solution of approx. 0.1 M PPh_3_ in C_6_D_6_ and are referenced to residual C_6_D_5_H and PPh_3_ (δ = −5.3) resonances, respectively. Samples for low‐temperature measurements incorporated an internal reference capillary containing a solution of approx. 0.1 M PPh_3_ in THF‐d_8_ and are referenced to residual THF‐d_7_. The paramagnetic susceptibility of samples was determined using the Evans NMR method [67]. An internal reference capillary containing C_6_H_6_ in THF‐d_8_ was used for variable temperature Evans measurements.

Infrared (IR) spectra were recorded using a PerkinElmer FT‐IR Spectrum GX spectrometer. Solutions were recorded in an airtight Specac Omni CellTM filled with 0.01‐0.015 M solutions via syringe in a glovebox. The optics, detector area, and sample chamber of the spectrometer were purged with dry N_2_ gas before each measurement to remove residual CO_2_ and H_2_O from the spectra. Samples measured as KBr pellets were combined with KBr (Sigma, FT‐IR grade) and ground to a powder, which was transferred into an airtight Specac die inside the glovebox. A Specac manual hydraulic press was then used outside the glovebox to press the pellet, which was then immediately measured to minimize sample decomposition.

Electronic spectra were recorded using a Perkin Elmer Lambda 20 UV‐visible spectrophotometer. Samples were prepared inside the glovebox using a quartz cuvette with an optical path length of 1 cm and fitted with a J. Young valve.

Electrochemical experiments were carried out using an AutoLab potentiostat controlled by Nova. Measurements were performed inside an Ar or N_2_ glovebox on room temperature solutions containing the sample (2 mM) and [*n*Bu_4_N][OTf] electrolyte (50 mM). A three‐electrode configuration was employed: a Pt working electrode (PWE) (BASi, Indiana, USA); a Pt wire counter electrode (99.99%; GoodFellow, Cambridge, UK); and an Ag wire pseudo‐reference electrode (99.99%; GoodFellow, Cambridge, UK). All electrodes were polished using alumina/H_2_O, and all electrodes were rinsed with Et_2_O and dried in a 100°C oven prior to each measurement. Measurements were calibrated to the ferrocene/ferrocenium couple (10 mM) at the end of each run, and iR was compensated to within 80 ± 5% of the solution's uncompensated resistance.

CW‐EPR X‐band measurements were performed with a Bruker‐Biospin Micro EMXplus spectrometer equipped with a PremiumX microwave bridge, a cylindrical TE011 resonator (SHQE‐W), an ESR‐900 liquid helium cryostat, and an Oxford Instruments ITC‐503s temperature controller.

Single crystal X‐ray diffraction measurements were performed with an Oxford Diffraction Xcalibur unit; crystals were mounted on a nylon MicroLoop using perfluoropolyether oil and measured in a stream of N_2_ at 173 K. Structures were solved in Olex2 [69] either by direct methods using the SHELXS solution program [70], charge flipping using Superflip [71], or charge flipping using the Olex2.solve structure solution program [72]. All data were subsequently refined with the ShelXL refinement package [73]. Deposition Number(s) 2475900 (for ***^i^* ^Bu^1**·N_2_) and 2475899 (for [***^i^* ^Bu^1**][BAr^F^_4_]) contain(s) the supplementary crystallographic data for this paper. These data are provided free of charge by the joint Cambridge Crystallographic Data Centre and Fachinformationszentrum Karlsruhe Access Structures service.

Elemental analyses were performed by Mr. S. Boyer of the London Metropolitan University.

MgBr_2_·2Et_2_O (Sigma‐Aldrich, 99%), FeCl_2_ (Sigma‐Aldrich, 98%), NaBH_4_ (Sigma‐Aldrich, 99%), 1, 2‐*bis*(dichlorophosphino)ethane (Sigma‐Aldrich, 97%), NH_4_Cl (VWR, 99.5%), [CoCp*_2_][PF_6_] (Alfa Aesar, 98%), and TfOH (Sigma‐Aldrich, 99%) were purchased and used without further purification. Cp_2_Fe was sublimed and recrystallized from pentane. Mg was heated at 140°C under high vacuum (1×10^−2^ mbar) for 12 h. Para‐dimethylaminobenzaldehyde (pdmab) was purified by recrystallization from cold EtOH. KC_8_ was prepared according to a literature procedure and ground into a fine powder using a mortar and pestle inside an Ar glovebox [74]. [Cp_2_Fe][BAr^F^_4_], [75] [Ph_2_NH_2_][BAr^F^_4_], [53] CoCp*_2_ [53, 76], 1, 2‐*bis*(diisobutylphosphino)ethane (dibpe), [77] and [*n*Bu_4_N][BAr^F^_4_] [78] were prepared according to literature procedures.

### 4.2 Synthesis of Fe(N_2_)(dibpe)_2_ (*^i^* ^Bu^1·N_2_)

FeCl_2_ (178 mg, 1.40 mmol) was added to THF (40 mL) in a Rotaflo ampoule and refluxed at 75°C until the solid dissolved. dibpe (892 mg, 2.80 mmol), MgBr_2_·2Et_2_O (181 mg, 0.70 mmol), and Mg (269 mg, 11.2 mmol) were then added successively; the reaction was stirred for 2 min between each addition. The ampoule was then frozen in a liquid N_2_ bath, the headspace evacuated, and backfilled with dry N_2_. The ampoule was thawed at 0°C behind a blast shield, such that a pressure of ∼4 bar N_2_ was achieved. The stirred reaction mixture was then allowed to warm to room temperature over the course of 2 h, and subsequently left stirring overnight. The pressure was released, and 1, 4‐dioxane (2 mL) was added. After stirring for a further 90 min, formation of a fine white precipitate was observed. The reaction mixture was filtered through a Celite pad on a sintered glass frit, and the solvent was removed in vacuo. The dark residue was extracted into pentane; this gave an orange solution, which was filtered 2x times through a Celite pad before being concentrated in a stream of N_2_ and left at ‐30°C overnight. Red crystalline ***^i^* ^Bu^1·N_2_** formed and was separated from the mother liquor, washed 2x with cold pentane, and dried under high vacuum (1×10^−2^ mbar). Crystals obtained by this method were suitable for x‐ray diffraction measurements. 470 mg of ***^i^* ^Bu^1·N_2_** was obtained (47%). (*Note: the mother liquor from crystallization typically contains significant quantities of dibpe, which can be recycled by following the distillation procedure described in the synthesis of the ligand [* *77* *])*.

Anal. Calcd. for C_36_H_80_FeN_2_P_4_: C, 59.99; H, 11.19; N, 3.89. Found: C, 59.84; H, 11.09; N, 3.80.

^1^H NMR (400.4 MHz, methylcyclohexane‐d_14_) δ / ppm: approx. 1.1 – 2.7 (br, m).

^31^P{^1^H} NMR (162 MHz, methylcyclohexane‐d_14_) δ / ppm: 79.7 (s).

IR (0.01 M Et_2_O solution): ν(^14^N‐^14^N) = 1984 cm^−1^.

### 4.3 Synthesis of Fe(^15^N_2_)(dibpe)_2_ (*^i^* ^Bu^1·^15^N_2_)

FeCl_2_ (36 mg, 0.28 mmol) was added to THF (40 mL) in a J. Young ampoule with side arm attachment and refluxed at 75°C until the solid dissolved. dibpe (178 mg, 0.56 mmol), MgBr_2_·2Et_2_O (36 mg, 0.14 mmol), and Mg (54 mg, 2.25 mmol) were added successively; the reaction was stirred for 2 min between each addition. The ampoule was then frozen in a liquid N_2_ bath, and a single freeze‐pump‐thaw cycle was performed. Under static vacuum, the ampoule was connected to a Toepler pump, and ^15^N_2_ was delivered. A pressure of ∼1 bar was obtained. The reaction and work‐up were then performed as detailed above, with all further manipulations carried out under Ar. Red crystalline ***^i^* ^Bu^1·N_2_** was obtained on recrystallization from pentane (42 mg, 21%).

^15^N NMR (40.55 MHz, pentane) δ / ppm: 331.1 (d, ^1^J_NN_ = 6.5 Hz), 330.8 (d, ^1^J_NN_  =  6.5 Hz).

IR (0.01 M Et_2_O solution): ν(^15^N‐^15^N) = 1899 cm^−1^.

### 4.4 Synthesis of [Fe(dibpe)_2_][BAr^F^_4_] ([*^i^* ^Bu^1][BAr^F^_4_])

[Cp_2_Fe][BAr^F^_4_] (69 mg, 0.07 mmol) was dissolved in Et_2_O (5 mL) and added dropwise to a stirred solution of ***^i^* ^Bu^1·N_2_** (50 mg, 0.07 mmol) in Et_2_O (5 mL) under Ar. An immediate color change to deep purple was observed, and the solution was stirred for 10 min. Pentane (10 mL) was added, and the solution slowly cooled to ‐30°C and maintained at that temperature overnight. The supernatant was decanted, and the purple solid was washed with pentane. The solid was then recrystallized from minimum Et_2_O at ‐30°C and again washed with pentane, then dried in vacuo. Purple crystalline **[*^i^* ^Bu^1][BAr^F^_4_]** was obtained (80 mg, 79%).

Anal. Calcd. for C_68_H_92_BFeF_24_P_4_: C, 52.48; H, 5.96. Found: C, 52.42; H, 5.93.

^1^H NMR (400.4 MHz, Et_2_O) δ / ppm: 7.70 (s, *meta*‐Ar^F^), 7.50 (s, *para*‐Ar^F^), −1.32 (br, s), −12.64 (br, s).

^11^B NMR (128.4 MHz, Et_2_O) δ: −6.6 (s).

^19^F NMR (376.4 MHz, CDCl_3_) δ: −62.4 (s).

UV‐VIS (THF, nm {m^2^ mol–^1^}): 269 {698}; 391 {130}; 520 {296}.

### 4.5 Acidification of *^i^* ^Bu^1·N_2_

Acidification reactions (with or without additional reductant) were performed, and their outcomes evaluated, in line with our previously published procedures [53, 54]. Reactions were performed using 0.008 mmol of ***^i^* ^Bu^1·N_2_** in 1.75 mL of solvent.

## Conflicts of Interest

The authors declare no conflict of interests.

## Supporting information




**Supporting File 1**: chem70489‐sup‐0001‐SuppMat.pdf.
