# Supplementary material for: Investigating N2 Fixation Using a Bulky Fe(bisphosphine)2 Framework
Source: Chemistry. 2025 Nov 28;32(1):e02688. doi: 10.1002/chem.202502688 (PMC12759171; doi:10.1002/chem.202502688)
Supplement: Supplementary file 1 — Supporting File 1: chem70489‐sup‐0001‐SuppMat.pdf. [file CHEM-32-e02688-s001.pdf]

Supporting Information

Investigating N_2 Fixation Using A Bulky Fe(bisphosphine)$_2$ Framework

Andrew D. Crawford,[a] Laurence R. Doyle,[a] Samuel J. Horsewill,[b] William K. Myers,[c] Daniel J. Scott,*[b] and Andrew E. Ashley*[a]

[a] Dr A. D. Crawford, Dr L. R. Doyle, Dr A. E. Ashley
Department of Chemistry, Imperial College London, Molecular Sciences Research Hub, London, W12 0BZ, UK.
E-mail: a.ashley@imperial.ac.uk

[b] Dr. S. J. Horsewill, Dr D. J. Scott
Department of Chemistry, University of Bath, Bath, BA2 7AY, UK.
E-mail: ds2630@bath.ac.uk

[c] Dr W. K. Myers
Inorganic Chemistry Laboratory, University of Oxford, Oxford, OX1 3QR, UK.

Table of contents

1. Spectral data for new Fe(dibpe)$_2$ complexes **S3**

1.1. Fe(N_2)(dibpe)$_2$ (^{iBu}**1·N$_2$**) and Fe($^{15}N_2$)(dibpe)$_2$ (^{iBu}**1·^{15}N$_2$**) S3

1.2. [Fe(dibpe)$_2$][BAr^F$_4$] ([^{iBu}**1**][BAr^F$_4$]) S7

1.3. Simulations of EPR spectra S10

1.4. DFT computations for EPR properties S12

2. Survey of N$_2$ reduction reactivity **S18**

2.1. Stoichiometric N_2 functionalisation S18

2.2. Attempted catalytic N_2 functionalisation S19

3. VT UV-vis analysis of N$_2$ binding to [^{iBu}1][BAr^F$_4$] **S20**

4. X-ray crystallographic data **S22**

4.1. Fe(N_2)(dibpe)$_2$ (^{iBu}**1·N$_2$**) S22

4.2. [Fe(dibpe)$_2$][BAr^F$_4$] ([^{iBu}**1**][BAr^F$_4$]) S24

5. References for supporting information **S26**

1. Spectral data for new Fe(dibpe)$_2$ complexes

1.1. Fe(N_2)(dibpe)$_2$ (^{iBu}**1·N$_2$**) and Fe($^{15}N_2$)(dibpe)$_2$ (^{iBu}**1·^{15}N$_2$**)

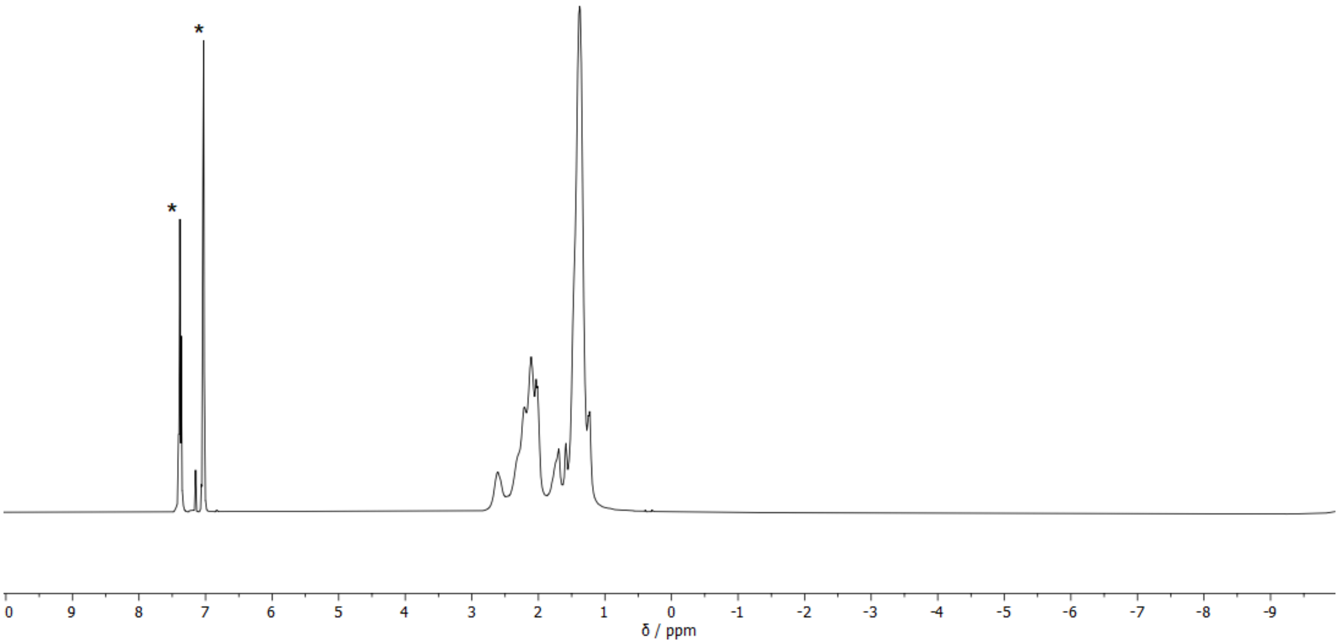

Figure S1. ^{1}H NMR spectrum of Fe(N_2)(dibpe)$_2$ (^{iBu}**1·N$_2$**) in methylcyclohexane-d$_{14}$.

(* Ph_3P in C_6D_6 capillary insert)

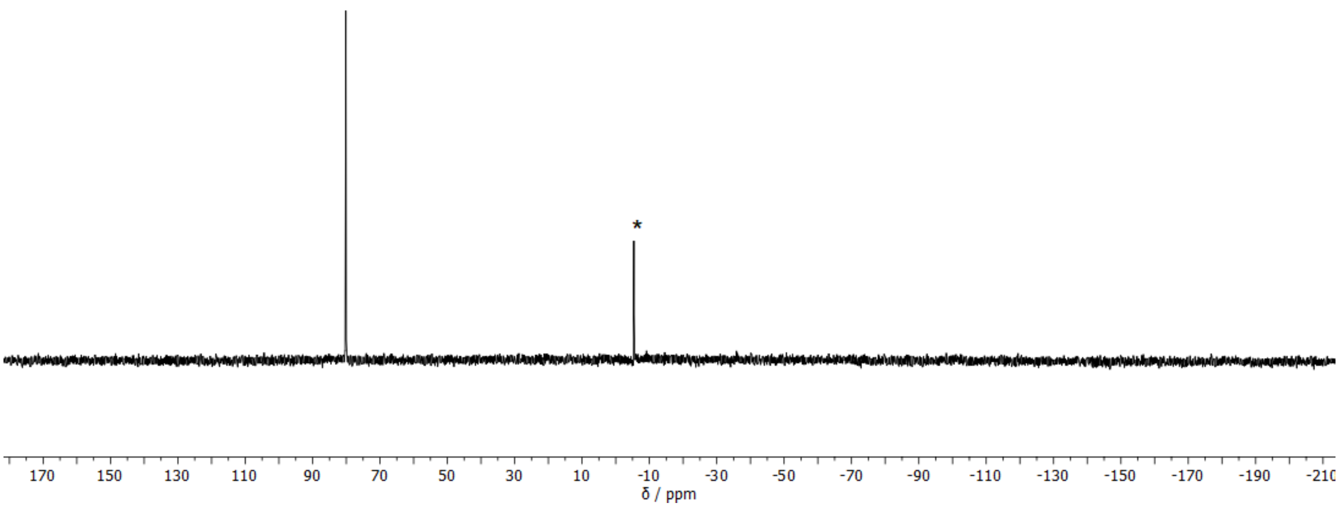

Figure S2. $^{31}P\{^1H\}$ NMR spectrum of $Fe(N_2)(dibpe)_2$ (^{iBu}**1·N$_2$**) in methylcyclohexane-d_{14}.

(* Ph_3P in C_6D_6 capillary insert)

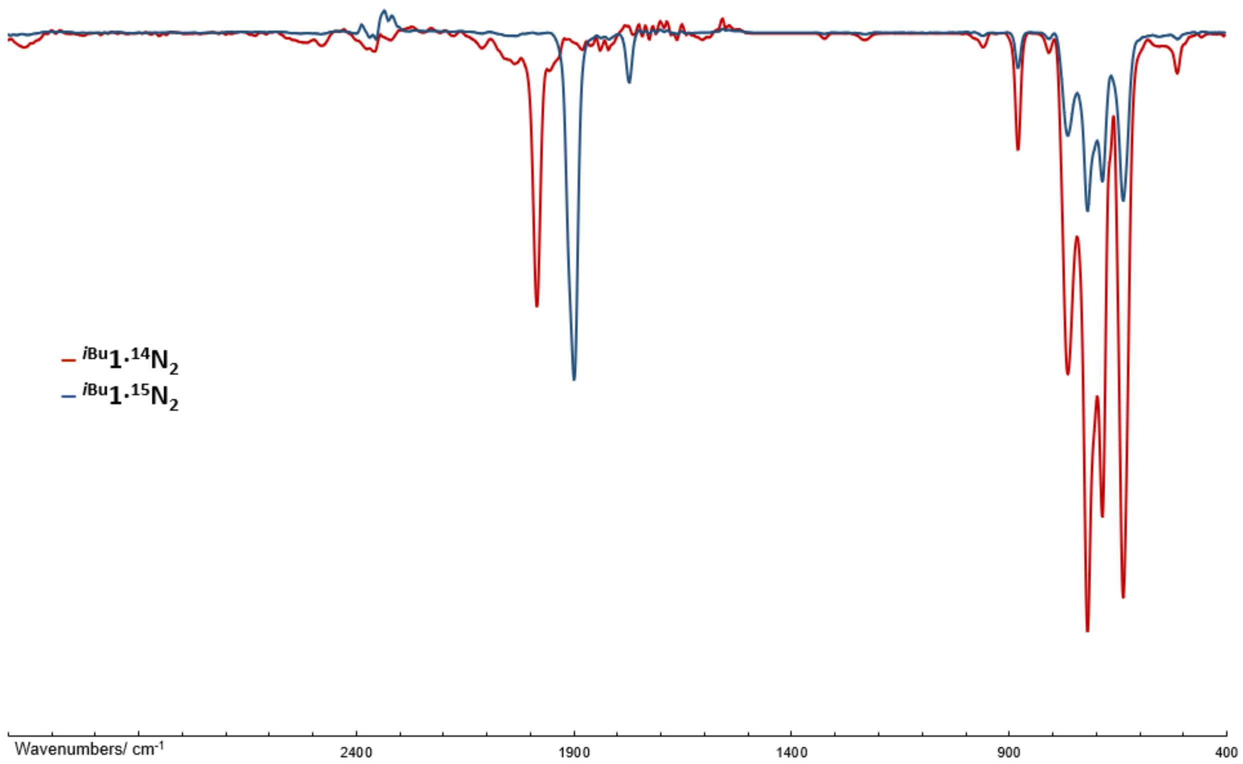

Figure S3. IR spectra of Fe($^{14/15}N_2$)(dibpe)$_2$ (^{iBu}**1·$^{14/15}$N$_2$**) in Et$_2$O (0.01 M).

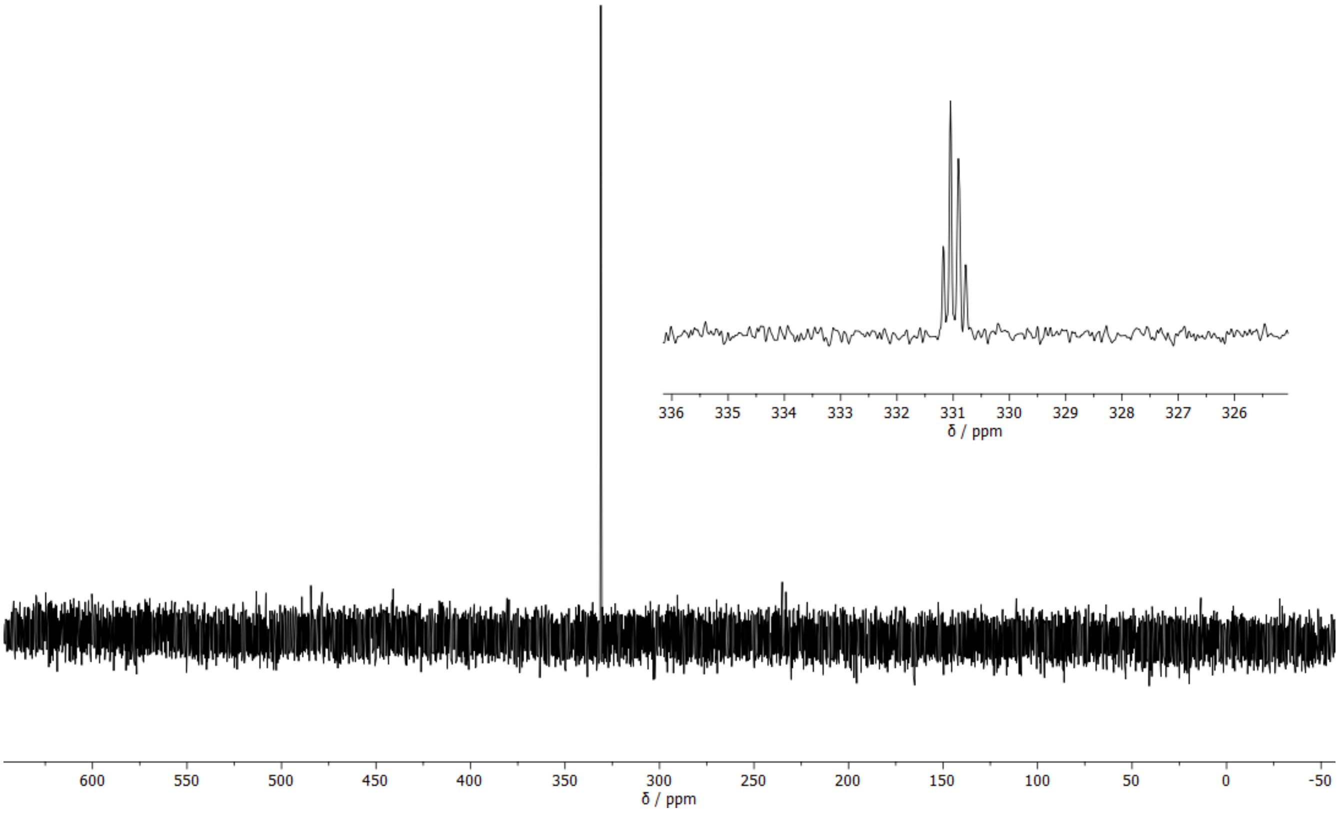

Figure S4. ^{15}N NMR spectra of $Fe(^{15}N_2)(dibpe)_2$ (^{iBu}**1·^{15}N$_2$**) in pentane.

1.2. [Fe(dibpe)$_2$][BAr$^F_4$] ([^{iBu}**1**][BAr$^F_4$])

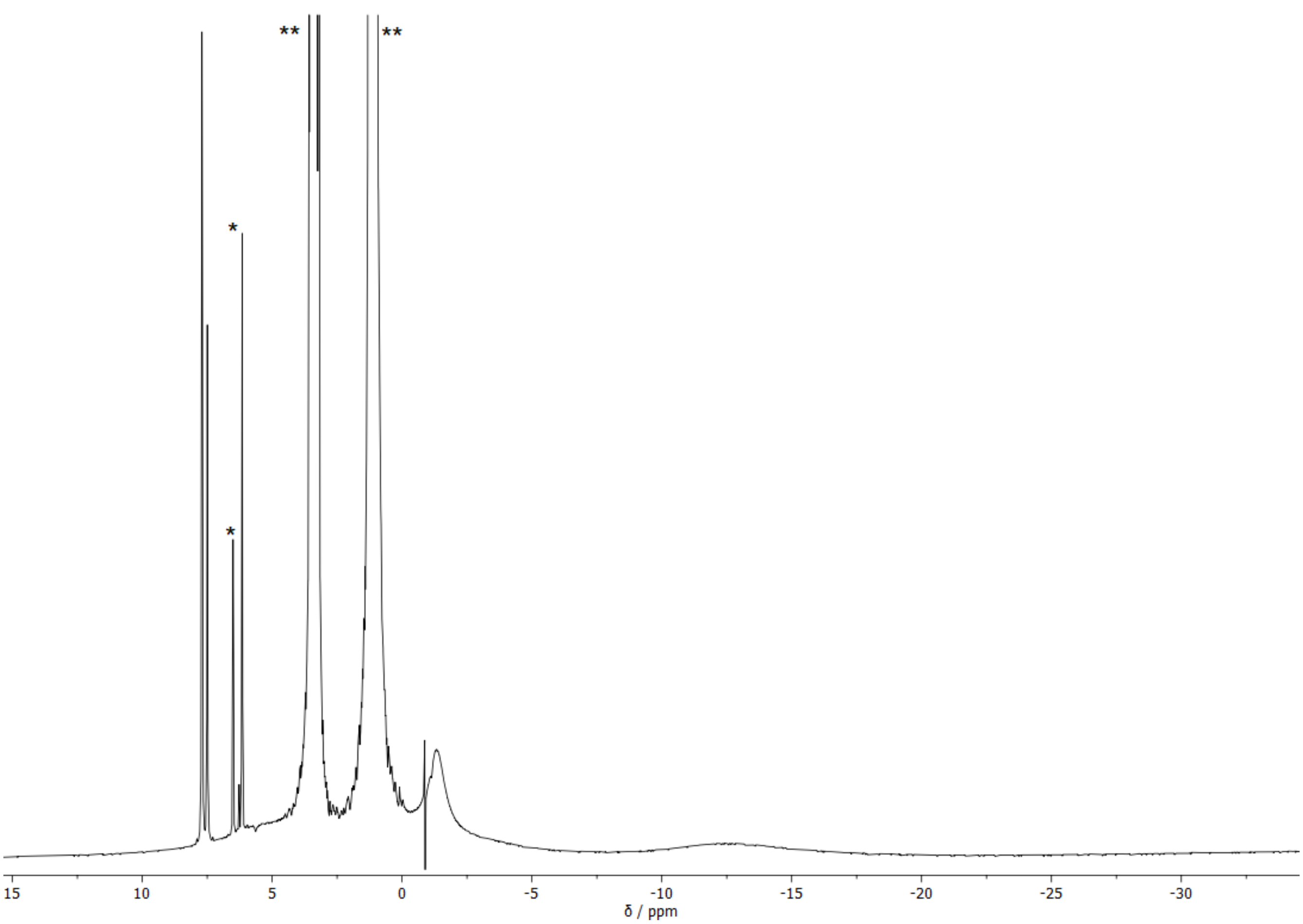

Figure S5. ^{1}H NMR spectrum of [Fe(dibpe)$_2$][BAr$^F_4$] ([^{iBu}**1**][BAr$^F_4$]) in Et_2O.

(* Ph_3P in C_6D_6 capillary insert; ** solvent truncated for clarity)

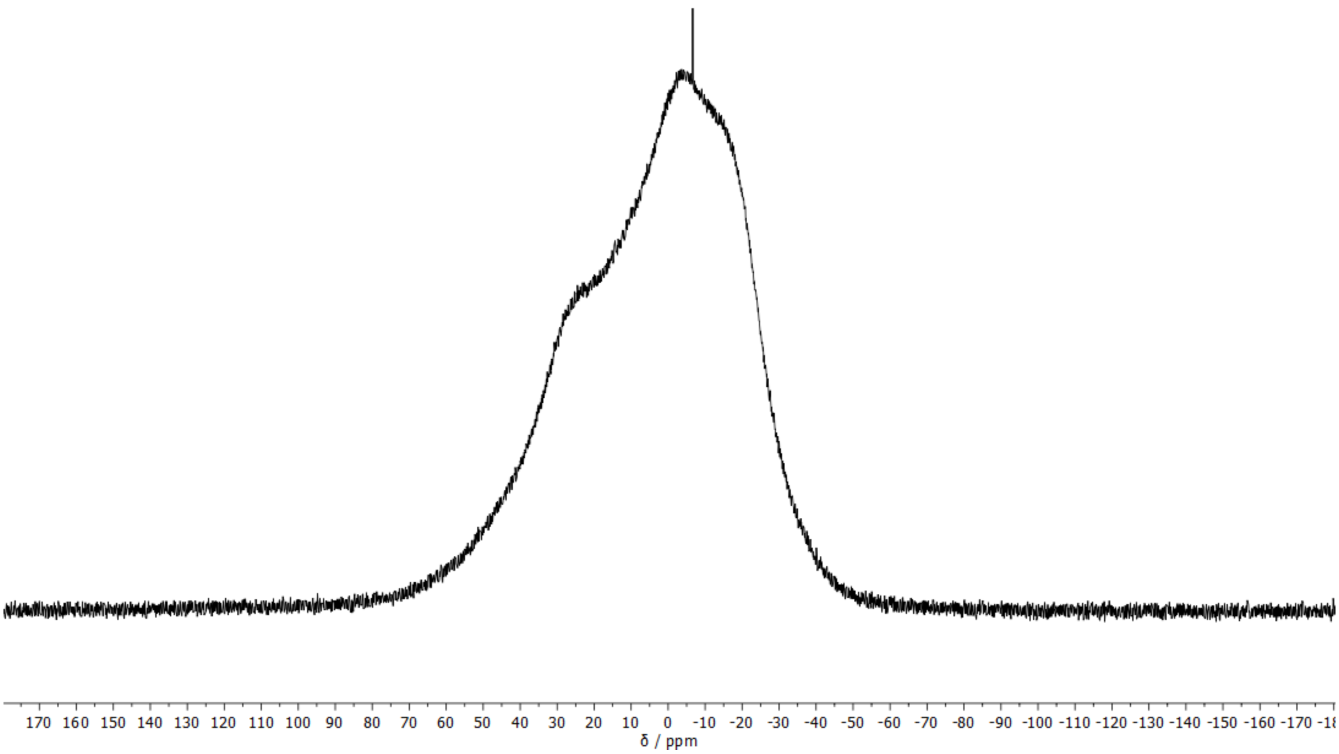

Figure S6. ^{11}B NMR spectrum of $[Fe(dibpe)_2][BAr^F{}_4]$ ($[^{iBu}\mathbf{1}][BAr^F{}_4]$) in Et_2O.

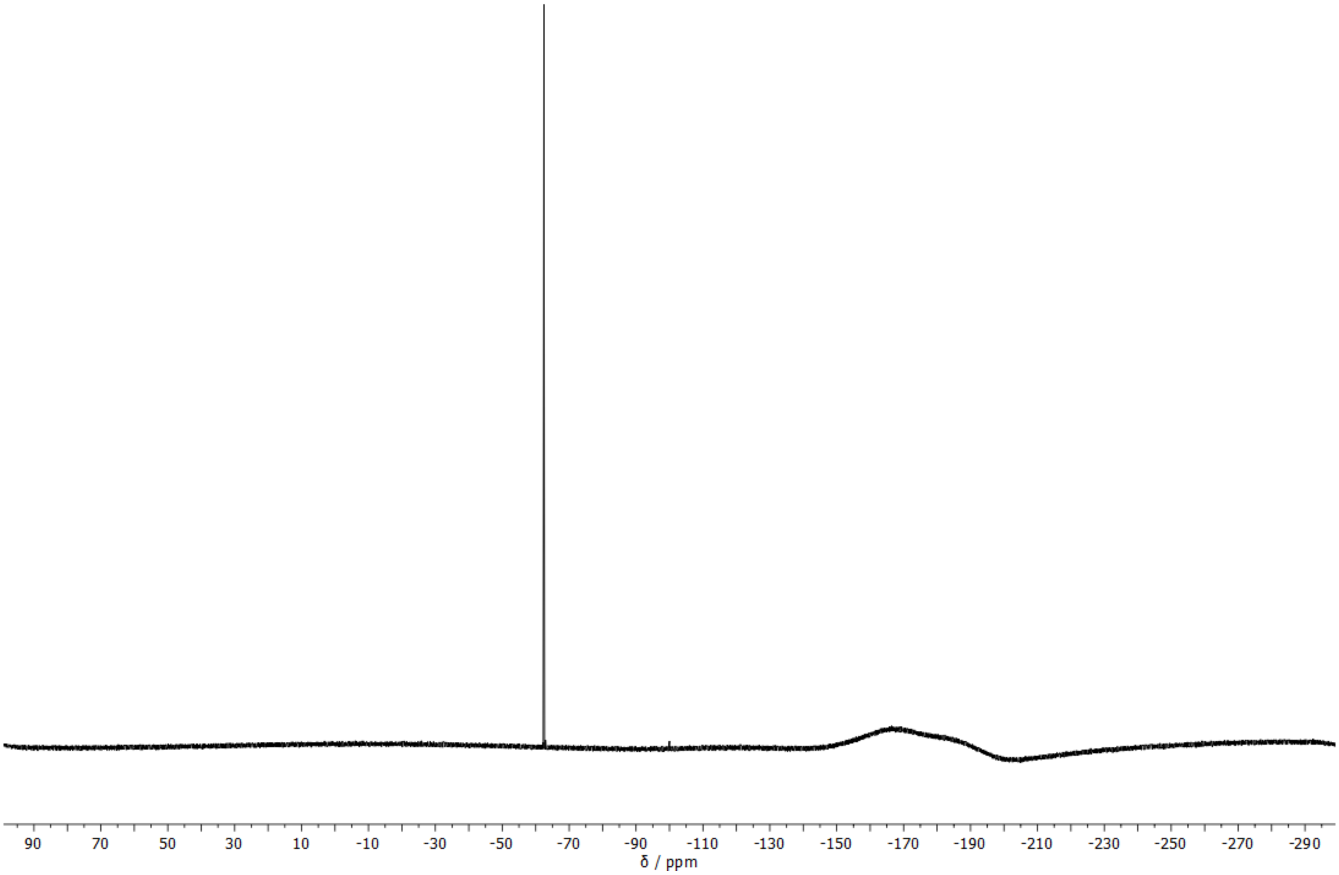

Figure S7. ^{19}F NMR spectrum of [Fe(dibpe)$_2$][BAr$^{F}{}_4$] ([$^{\boldsymbol{i}\mathbf{Bu}}$**1**][BAr$^{F}{}_4$]) in Et_2O.

1.2. Simulations of EPR spectra

Using the EPR simulation codes of Easyspin 6.0.6[34], CW-EPR data collected on ${}^{iBu}\mathbf{1}\cdot N_2^+$ and $[{}^{iBu}\mathbf{1}]^+$ could be simulated with satisfactory fits, shown in Figure S8. In the absence of complete field-dependent ENDOR studies, the relative orientations of the g-matrix and hyperfine tensors of the first coordination sphere were taken from a DFT calculation using Orca(35-50), shown in Table S1. For both complexes the g-matrix required adjustment, which is not unexpected for transition metals and might be improved with an expanded basis. The A_Z values ${}^{iBu}\mathbf{1}\cdot N_2^+$ required reduction of 0.85 of their value, as an approximation, at g_{min}. However, for the $[{}^{iBu}\mathbf{1}]^+$ at EPR resolution, ^{31}P hyperfine matched DFT without additional modification along the $g_{z,min}$.

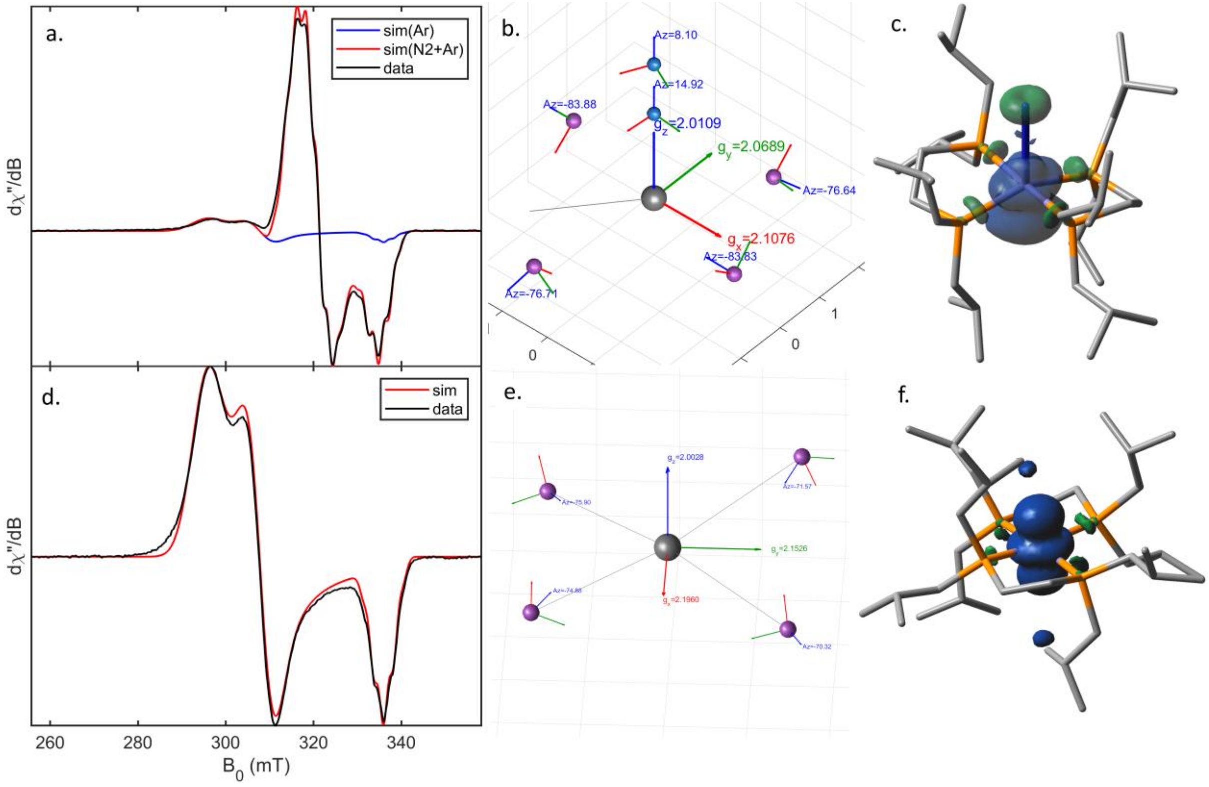

Figure S8. X-band EPR, tensor orientations, and spin density (contour at 0.002 e/au^3) plots (a., b., c.) of ${}^{iBu}\mathbf{1}\cdot N_2^+$ and (d., e., f.) of $[{}^{iBu}\mathbf{1}]^+$. The data was acquired at 40 K, with ν_{mw} = 9.3906 GHz (a) and ν_{mw} = 9.3109 GHz (d), for both samples, microwave power was 50.24 uW, field modulation was 0.5 mT. Simulation values for panel a., principal g-values g_1 = 2.129, g_2 = 2.809, g_3 = 2.0038, hyperfine couplings of Table S1 are multiplied by Az*0.85 to fit data, linewidths were $l.w._{1,3}$ 60 MHz and $l.w._2$ 53 MHz. Simulation values for panel d., principal g-values g_1 = 2.27, g_2 = 2.18, g_3 = 1.997, hyperfine couplings of Table S1 are used as is, linewidths were $l.w._1$ = 196 MHz, $l.w._2$ = 120 MHz, and $l.w._3$ = 60 MHz.

Table S1. EPR DFT parameter values.

		$^{iBu}\mathbf{1}\cdot N_2^+$							$^{iBu}[\mathbf{1}]^+$				
	value	g	^{31}P	^{31}P	^{31}P	^{31}P	^{14}N	^{14}N	g	^{31}P	^{31}P	^{31}P	^{31}P
Prin-cipal value	1	2.1076	-55.5	-65.0	-55.4	-64.9	12.2	0.8	2.1960	-59.2	-62.6	-61.6	-57.8
	2	2.0689	-61.4	-66.3	-61.3	-66.3	12.5	1.8	2.1526	-60.0	-63.7	-62.6	-58.6
	3	2.0109	-76.7	-83.9	-76.6	-83.8	14.9	8.1	2.0028	-71.6	-75.9	-74.9	-70.3
Z-Y-Z Euler angle	α	0	-119	179	61	179	22	24	0	-25	22	152	23
	β	0	130	91	130	89	0	0	0	106	75	85	84
	γ	0	-19	50	161	130	-117	-140	0	15	-171	-176	-176

1.4. DFT computations for EPR properties

Computations started from the crystallographic cation coordinates and these were input into Orca 6.1.0 package[35-50] for geometry optimisation with the TightOpt keyword, single point energy, and EPR properties output. The B3LYP functional was used with a basis choice per element: Fe "def2-TZVPP", P & N "def2-TZVP", and C & H "def2-SVP". Visualisation of results are generated via custom scripts in Matlab R2024b (Mathworks, Natick, NJ) and GaussView 6.0.16 (Gaussian, Inc.).

Coordinates of ^{iBu}**1**·N_2^+ DFT calculations (fig. S1c)
Final single point energy = -4156.341311008945 a.u.

Fe	0.001958383	0.002846196	-0.219503305
P	-2.285545265	0.006267843	-0.749261006
P	-0.234985344	2.321449786	-0.206817479
P	2.289052321	-0.000890698	-0.751734900
P	0.239081026	-2.316171244	-0.205321758
N	0.003627032	0.003074699	1.672116925
N	0.005107786	0.002719643	2.783002546
C	-2.858595782	1.771153138	-0.871010083
C	-2.034013800	2.660124465	0.061929663
C	-3.520598460	-0.817826446	0.378194126
C	-3.683132461	-0.372462566	1.853533250
C	-4.175273986	-1.557865490	2.701437271
C	-4.648196296	0.811328603	2.022131731
C	-2.908176697	-0.746934157	-2.334195957
C	-2.086264395	-0.637135772	-3.636679819
C	-2.119712768	0.759085355	-4.273127520
C	-2.581873459	-1.685062464	-4.646921885
C	0.206051592	3.284747535	-1.729135123
C	-0.124834874	4.799837739	-1.824346670
C	1.023983628	5.556356259	-2.510899732
C	-1.445888519	5.060384212	-2.563758990
C	0.680154460	3.257383382	1.123846755
C	-0.032608975	4.333670569	1.988068077
C	-0.719513533	3.724885899	3.219442122
C	0.976260150	5.408417132	2.423585935
C	2.862255376	-1.765925437	-0.871366867
C	2.038295959	-2.653690358	0.063251794
C	3.525387999	0.825403884	0.372708252
C	3.690339842	0.382942398	1.848619343
C	4.185329280	1.569747493	2.692910565
C	4.654531378	-0.801464622	2.017941561
C	2.909817978	0.749889516	-2.338611596
C	2.086446727	0.638279810	-3.640004306
C	2.119405023	-0.758803775	-4.274559538
C	2.580800362	1.684911261	-4.652205645
C	-0.202671790	-3.280969338	-1.726452655
C	0.128230045	-4.796072028	-1.821037052

C	-1.020128401	-5.552534370	-2.508434983
C	1.449820025	-5.056988522	-2.559357822
C	-0.675185117	-3.250560092	1.126832490
C	0.036967895	-4.328745521	1.989302125
C	0.726065789	-3.722048016	3.220478942
C	-0.972962044	-5.402453061	2.424912196
H	-2.724609555	2.086129035	-1.915302967
H	-3.936388519	1.841299631	-0.659896453
H	-2.247821205	2.416782759	1.112130018
H	-2.273548094	3.724774984	-0.072938999
H	-4.502137829	-0.777972236	-0.127103997
H	-3.215777859	-1.876703222	0.357207127
H	-2.696632540	-0.073785363	2.246674944
H	-4.299228284	-1.264807768	3.755952252
H	-5.152196028	-1.922354872	2.340385488
H	-3.470988635	-2.404546865	2.669794817
H	-4.755202548	1.073467330	3.086604185
H	-4.318418367	1.718923306	1.496735528
H	-5.652493190	0.554038794	1.644246518
H	-3.042180924	-1.814292032	-2.093571091
H	-3.927940454	-0.354185126	-2.498661174
H	-1.034326558	-0.872633710	-3.394594538
H	-1.578241749	0.760985813	-5.232264173
H	-3.155311521	1.078990147	-4.479793141
H	-1.651313898	1.525420018	-3.638391162
H	-1.997482967	-1.644496935	-5.580066326
H	-2.503885744	-2.708632307	-4.246064779
H	-3.638901207	-1.510667164	-4.910317625
H	1.295052785	3.140370493	-1.809209187
H	-0.214906290	2.747809110	-2.593809109
H	-0.218969056	5.211128071	-0.804132700
H	0.799962604	6.632148449	-2.589178079
H	1.970112212	5.451842615	-1.954851513
H	1.191589162	5.176740470	-3.533518744
H	-1.676886250	6.137122008	-2.586125986
H	-1.381678494	4.715271911	-3.609999762
H	-2.303135537	4.550663592	-2.098050056
H	1.527736590	3.720511168	0.592781406
H	-0.802288769	4.836551764	1.377932425
H	-1.445254698	2.939056065	2.960175544
H	0.024229708	3.270374538	3.895575019
H	-1.258635301	4.497833293	3.789579787
H	0.492808300	6.172753665	3.052589469
H	1.796640747	4.964455910	3.013188537
H	1.424624903	5.922009830	1.557513223
H	2.727779517	-2.082346202	-1.915166634
H	3.940157530	-1.835619927	-0.660684308
H	2.252271940	-2.408461754	1.112992715
H	2.278237183	-3.718478483	-0.069834312
H	4.506252180	0.785050930	-0.133862865

H	3.220125272	1.884132540	0.350305760
H	2.704324855	0.086124029	2.244348672
H	4.310892730	1.278937229	3.747855908
H	5.161973824	1.932431822	2.329294356
H	3.481872045	2.417100128	2.660630977
H	4.764534676	-1.060321171	3.082911651
H	4.321960230	-1.710355950	1.496565238
H	5.657965643	-0.546591126	1.636142907
H	3.044278145	1.817581664	-2.099764070
H	3.929333768	0.356731472	-2.503632265
H	1.034744032	0.873986547	-3.397092014
H	1.576631609	-0.762203106	-5.232955363
H	3.154812559	-1.078713958	-4.482166369
H	1.652095403	-1.524347926	-3.638074413
H	1.995395118	1.643003862	-5.584654881
H	2.503109160	2.709010716	-4.252643031
H	3.637560981	1.510304475	-4.916535969
H	-1.291766390	-3.136812615	-1.805727291
H	0.217458391	-2.744641623	-2.591903922
H	0.221435263	-5.207146395	-0.800671571
H	-0.796138052	-6.628351917	-2.586441476
H	-1.966687135	-5.447891468	-1.953147368
H	-1.186875394	-5.172994791	-3.531222394
H	1.680532340	-6.133792356	-2.581457056
H	1.386521672	-4.711960425	-3.605682168
H	2.306854054	-4.547514141	-2.093012948
H	-1.117534248	-2.499157695	1.798041982
H	-1.524756002	-3.711731439	0.597204021
H	0.805306601	-4.832153336	1.377925563
H	1.452852486	-2.937233507	2.961087157
H	-0.016239975	-3.266851754	3.897734796
H	1.264493017	-4.496333652	3.789460047
H	-0.489955179	-6.168044115	3.052734404
H	-1.792104749	-4.957865618	3.015765509
H	-1.422964711	-5.914642945	1.558854996
H	1.125209578	2.506575971	1.793950320

Coordinates of ^{iBu}[**1**]$^{+}$ DFT calculations (fig. S1f)
Final single point energy = -4046.821384200659 a.u.

Fe	-0.001508007	0.037307894	0.187807971
P	1.741676551	1.556402410	0.018085162
P	1.658554133	-1.554022098	0.142901891
P	-1.763586451	-1.458188889	0.004637182
P	-1.698031170	1.591317051	0.565301008
C	3.375990017	0.663190146	0.089813749
C	3.248634487	-0.776538651	-0.412598820
C	-3.367953436	-0.528980729	-0.070058287
C	-3.297490337	0.690257309	0.844210125

C	1.925258478	2.928023791	1.261539357
C	2.934014764	4.093742236	1.117144122
C	4.406835937	3.678066981	1.225951826
C	2.609224915	5.165994522	2.171925367
C	1.857975721	2.484132909	-1.590097353
C	1.915524675	1.664347392	-2.894357396
C	0.636741343	0.859382172	-3.143191954
C	2.222628691	2.585365157	-4.085313262
C	1.569478908	-3.102404834	-0.878749478
C	2.820727711	-4.012297437	-1.040341175
C	3.564075811	-3.742936744	-2.357249647
C	2.423599689	-5.494136955	-0.945506452
C	2.849371766	-2.155710304	4.195859282
C	0.978563059	-0.657631874	3.399642184
C	-1.974862020	-2.703503008	-1.362812021
C	-1.593267284	-2.336534355	-2.813704933
C	-1.597744272	-3.607464505	-3.678638249
C	-2.491344990	-1.262649750	-3.442543550
C	-1.942225029	-2.558652158	1.494937411
C	-3.224778254	-3.413269233	1.695449392
C	-2.864471640	-4.832047993	2.163337184
C	-4.201115777	-2.754956847	2.681683870
C	-1.735099334	2.866162400	1.923336612
C	-1.213038859	2.522035093	3.335154400
C	-1.139651970	3.805820621	4.177799221
C	-3.339207386	3.541857447	-0.958661619
C	-4.429509923	2.887772343	-1.820324692
C	2.084118575	-2.300816884	1.795400126
C	2.276732175	-1.368369472	3.007076642
C	-2.041189768	1.455462076	4.064160482
C	-2.048300514	2.677247288	-0.906109977
C	-3.025525376	4.955774941	-1.472484355
H	4.141292991	1.208721507	-0.481431349
H	3.699104685	0.679702993	1.141360451
H	4.115752117	-1.384859429	-0.111216491
H	3.218127777	-0.789633108	-1.512885787
H	-3.509807117	-0.220316403	-1.115392809
H	-4.213634746	-1.187650060	0.178993925
H	-3.312173446	0.382379279	1.899025183
H	-4.155452087	1.365159985	0.703979373
H	0.917136141	3.362553472	1.326780402
H	2.082820051	2.420418203	2.230018438
H	2.783902460	4.560768660	0.127664993
H	5.056856408	4.567167164	1.228755778
H	4.734883986	3.043377522	0.390742699
H	4.598530473	3.129280465	2.164202494
H	2.737142626	4.769522938	3.193756827
H	1.572212343	5.528086942	2.080557887
H	3.276473429	6.036019448	2.067053684
H	0.998022048	3.175084736	-1.625222462

H	2.758224737	3.115437118	-1.525344463
H	2.757065550	0.952296987	-2.811956401
H	0.400333100	0.190947492	-2.293450723
H	0.729923770	0.230021387	-4.041939366
H	-0.225966354	1.528361328	-3.294221141
H	1.426635765	3.337606767	-4.220578486
H	2.299343581	2.009883267	-5.021680427
H	3.172538411	3.125698814	-3.945320070
H	0.753573129	-3.678837232	-0.414230419
H	1.192916414	-2.804015112	-1.869965042
H	3.523937891	-3.814243201	-0.212248642
H	2.919941357	-3.970603693	-3.223910174
H	4.461354830	-4.376843966	-2.437334583
H	3.891707798	-2.696517639	-2.451117120
H	1.960908367	-5.729925730	0.026910229
H	3.301574479	-6.148834423	-1.065068267
H	1.700905461	-5.760725819	-1.735899401
H	3.817310431	-2.619250988	3.946573007
H	2.162231323	-2.961820712	4.505231063
H	0.218480720	-1.384547494	3.729767060
H	0.552049307	-0.085326444	2.552981295
H	-1.377920304	-3.576044124	-1.054751682
H	-3.026303647	-3.039005724	-1.337961561
H	-0.561942241	-1.943029952	-2.803288346
H	-0.911285699	-4.373011941	-3.281588855
H	-1.290842326	-3.384241720	-4.712937150
H	-2.605659141	-4.054335154	-3.720642753
H	-3.552888521	-1.562907480	-3.416903093
H	-2.221065011	-1.102780279	-4.498183896
H	-2.400431446	-0.289213439	-2.937243082
H	-1.058421754	-3.215583338	1.436513535
H	-1.766588706	-1.924601776	2.378784275
H	-3.745484602	-3.517081187	0.727102720
H	-3.769074623	-5.444437949	2.306699240
H	-2.324563714	-4.807021243	3.125501590
H	-5.124775486	-3.347579834	2.777028923
H	-4.491813460	-1.738873917	2.371665360
H	-1.156843918	3.720287130	1.534302681
H	-2.775847134	3.226496180	1.998756420
H	-0.186134908	2.132988116	3.230281704
H	-2.141071627	4.248016440	4.315560640
H	-0.501062789	4.568949671	3.704191773
H	-0.727970409	3.598991027	5.178558113
H	-1.993044916	0.473798446	3.568682244
H	-3.741677765	3.652914916	0.063650348
H	-5.355688803	3.483943128	-1.804507358
H	-4.684432102	1.872819503	-1.477315689
H	1.278314305	-3.020937688	2.021726487
H	2.996666129	-2.900272112	1.645429697
H	3.022632303	-0.598825127	2.739229159

H	-1.670027450	1.314844270	5.091545564
H	-1.158153454	3.324757868	-0.965534996
H	-2.301542462	5.473177798	-0.821820623
H	-3.936515563	5.573969340	-1.515506609
H	3.005987167	-1.499776420	5.067091781
H	1.142739744	0.050796209	4.226285638
H	-1.988799612	2.031078252	-1.796379027
H	-2.598887881	4.921436602	-2.489645747
H	-4.103635517	2.811045410	-2.871910481
H	-3.102035391	1.750736894	4.135616214
H	-2.223444083	-5.350181384	1.431296901
H	-3.751054996	-2.679688895	3.686521425

2. Survey of N_2 reduction reactivity

2.1. Stoichiometric N_2 functionalisation

Table S2. Results of acidification (24 eq. acid) of compounds **^{R}1·N$_2$.**

Entry	**^{R}1·N$_2$** R =	T / °C	Solvent	Acid	NH_3 / %[a]	N_2H_4 / %[a]	e⁻ yield / %[b]
1[c]	***i*Bu**	20	pentane	TfOH	14	16	51.3
2[d]	Et	20	pentane	TfOH	8	21	53.5
3[c]	***i*Bu**	20	Et_2O	TfOH	25	3	42.3
4[d]	Et	20	Et_2O	TfOH	6	11	31.5
5	***i*Bu**	–78	pentane	TfOH	3	6	15.3
6[d]	Et	–78	pentane	TfOH	5	24	54.8
7	***i*Bu**	20	Et_2O	[Ph_2NH_2]OTf	5	2	11.5
8[e]	Et	20	Et_2O	[Ph_2NH_2]OTf	26	1	39

[a] Yields per mol Fe. [b] Yield assuming each Fe supplies a max. of two electrons. [c] Average of two runs. [d] Values taken from ref. 13e. [e] Values taken from ref. 13d.

Previously, excellent results were obtained by treating **^{Et}1·N$_2$** with the strong acid TfOH in pentane at ambient temperature (20 °C);[13e] indeed, acidification of **^{iBu}1·N$_2$** using an identical protocol led to the formation of significant quantities of both NH_3 and N_2H_4 (Table S2, entry 1). Notably, the efficiency of this reaction, as defined by the electron yield, is very similar to when using **^{Et}1·N$_2$** (Table S2, entry 2), making **^{iBu}1·N$_2$** one of the most efficient known compounds for stoichiometric azane production.

Inferior results were obtained when the solvent was switched from pentane to Et_2O; again, this is consistent with results obtained for **^{Et}1·N$_2$** (Table S2, entries 3-4). However, some differences were observed; both upon switching to lower temperature (which led to significantly reduced production of fixed N for **^{iBu}1·N$_2$**, but not for **^{Et}1·N$_2$**; Table S2, entries 5-6), and upon switching to a weaker acid (which led to poorer results for **^{iBu}1·N$_2$**, but slightly improved results for **^{Et}1·N$_2$**; Table S2, entries 7-8). From these results we speculate that, in addition to the anticipated slowing of unproductive protonation at Fe, the increased steric bulk of **^{iBu}1·N$_2$** may also appreciably slow the rate of the desired N_2 reduction reaction relative to **^{Et}1·N$_2$**, particularly at low temperature or when using less powerful (and bulkier) acids, hence reducing product yield in these cases (differences in relative rates of decomposition of the Fe(PP)$_2$ motifs may also be possible).

2.2. Attempted catalytic N_2 functionalisation

Table S3. Results of stoichiometric and attempted catalytic acidifications of ***i*Bu1·N₂**, and selected analogous reactions using less bulky **Et1·N₂** and **Me1·N₂** highlighting the much poorer performance of the latter relative to the former.[a]

Entry	^{R}**1·N$_2$** R =	T / °C	Solvent	Acid	Reductant	NH_3 / %[b]	N_2H_4 / %[b]	e⁻ yield / %[c]
1[d,e]	*i*Bu	20	pentane	TfOH	-	14	16	51.3
2[d,e]	*i*Bu	20	Et_2O	TfOH	-	25	3	42.3
3[d]	*i*Bu	20	THF	TfOH	-	19	1	28.9
4[e]	*i*Bu	–78	pentane	TfOH	-	3	6	15.3
5	*i*Bu	–78	Et_2O	TfOH	-	8	10	31.6
6	*i*Bu	–78	THF	TfOH	-	10	9	31.8
7	*i*Bu	–78	THF	TfOH	$CoCp^*_2$	97	2	15.0
8	*i*Bu	20	THF	TfOH	$CoCp^*_2$	77	0	11.5
9	*i*Bu	–78	pentane	TfOH	$CoCp^*_2$	22	0	3.3
10	*i*Bu	20	Et_2O	$[Ph_2NH_2][OTf]$	-	5	2	11.5
11	*i*Bu	–78	Et_2O	$[Ph_2NH_2][OTf]$	-	0	2	4.6
12	*i*Bu	–78	Et_2O	$[Ph_2NH_2][BAr^F_4]$	-	14	7	35.2
13	*i*Bu	–78	Et_2O	$[Ph_2NH_2][OTf]$	$CoCp^*_2$	156	1	23.6
14	*i*Bu	–78	Et_2O	$[Ph_2NH_2][BAr^F_4]$	$CoCp^*_2$	0	0	0
15	*i*Bu	–78	Et_2O	$[Ph_2NH_2][BAr^F_4]$	$CoCp^*_2$	54	0	8.1
16	Et	–78	THF	TfOH	$CoCp^*_2$	109	136	43.6
17	Me	–78	THF	TfOH	$CoCp^*_2$	2	44	9.1

[a] Acidification reactions (with or without additional reductant) were performed, and their outcomes evaluated, in line with our previously published procedures.[13d,13e] Reactions were performed using 0.008 mmol of ^{R}**1·N$_2$**, 24 eq. of acid, and 18 eq. of reductant in 1.75 mL of solvent. [b] Yields per mol Fe. [c] Yield assuming each Fe supplies a max. of two electrons. [d] Average of two runs. [e] Values reproduced from Table S2.

3. VT UV-vis analysis of N_2 binding to [^{iBu}**1**][$BAr^F{}_4$]

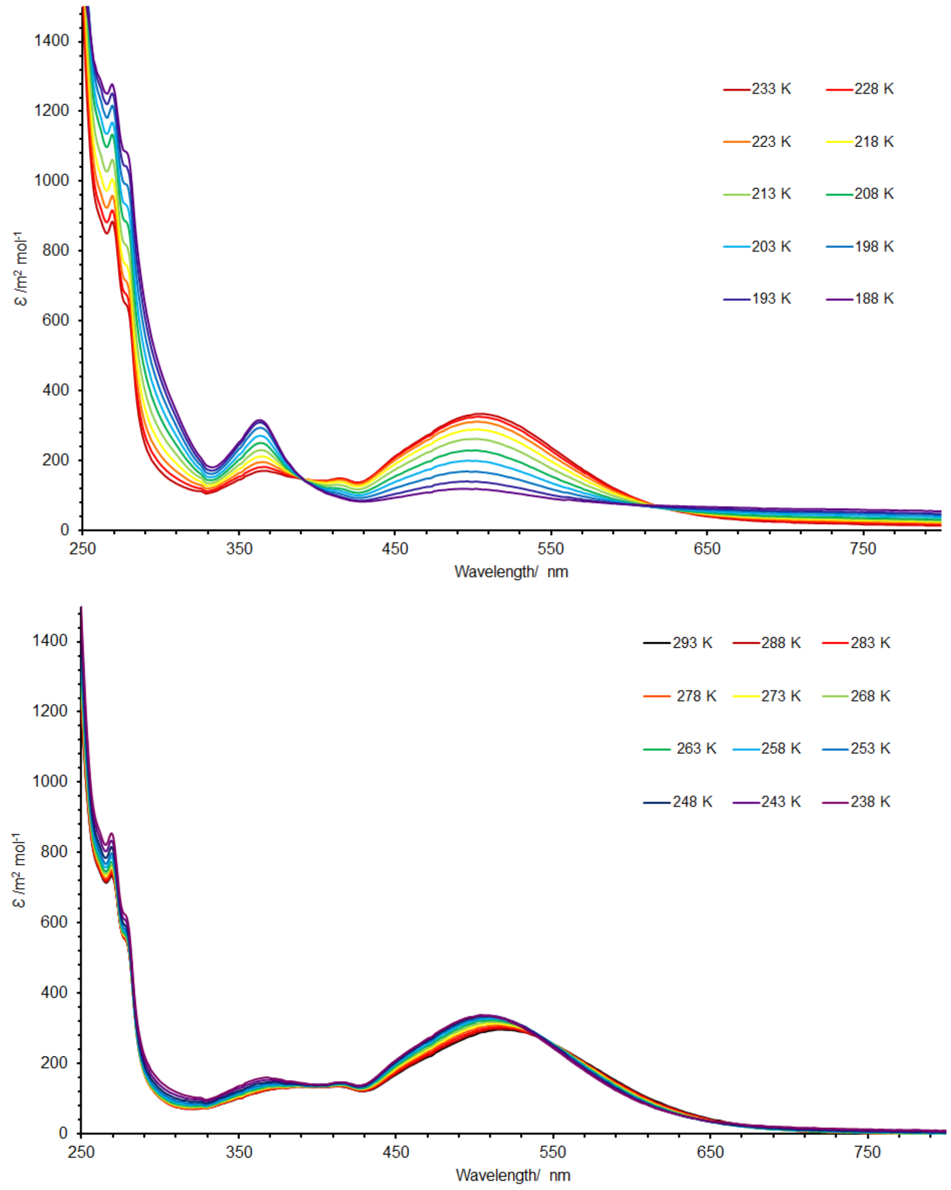

Figure S10. VT UV-vis spectra of [^{iBu}**1**][$BAr^F{}_4$] as 0.15 mM THF solution under 1 atm N_2. Top: Base-free regime between 293 and 238 K where no N_2 binding is observed. Bottom: Decrease in intensity of the major absorption and appearance of a new feature at 365 nm occurring below 233K, associated with N_2 binding.

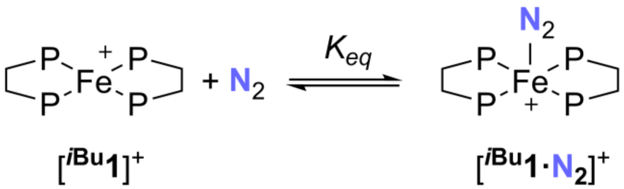

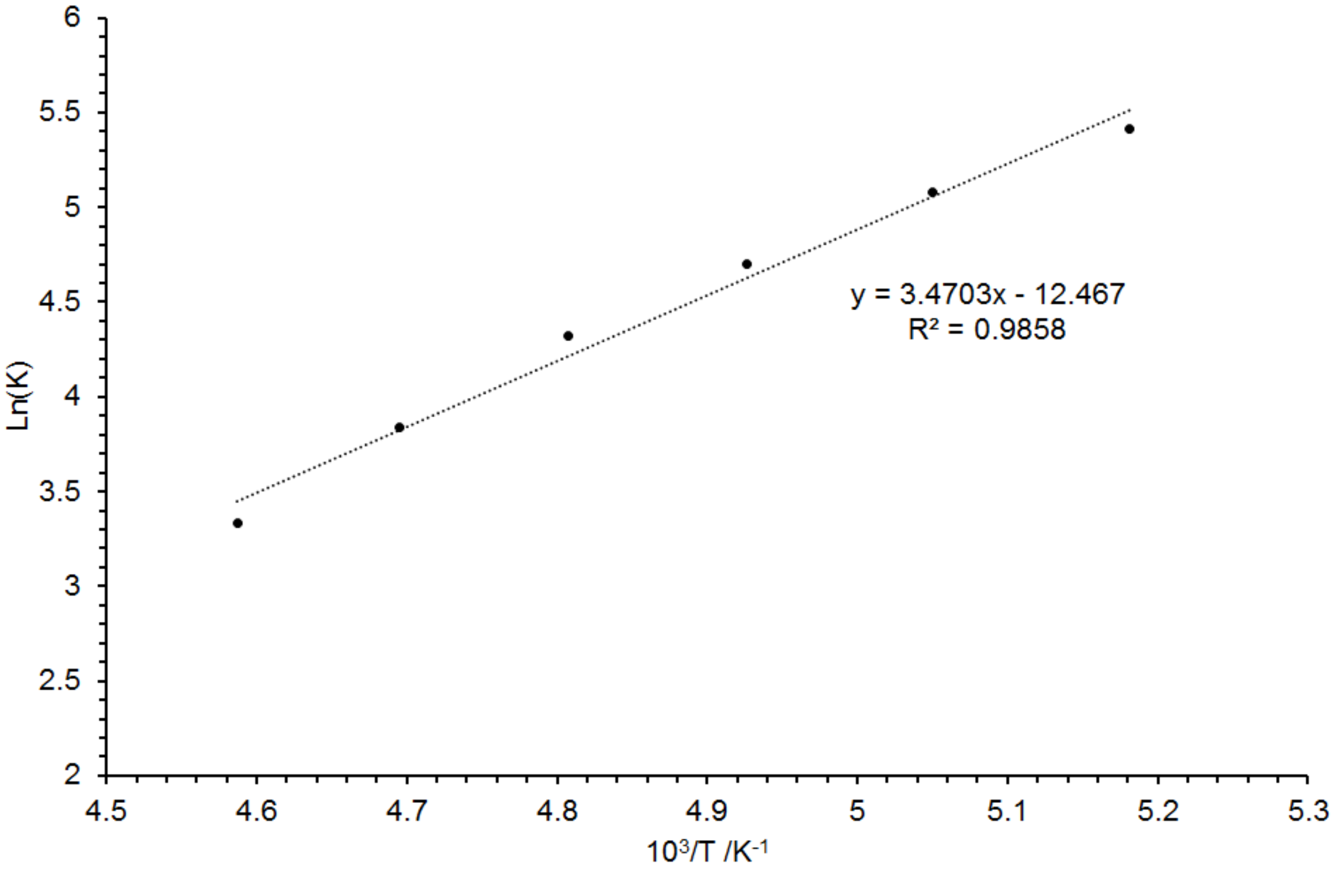

$\ln K = -\Delta H/RT + \Delta S/R$ $y = mx + c$

$m = -\Delta H/(R \times 10^3)$ $c = \Delta S/R$ $R = 1.987$ cal mol^{-1} K^{-1}

ΔH / kcal mol^{-1} = -6.92 (std. 0.51)

ΔS / cal K^{-1} mol^{-1} = -25.1 (std. 2.48)

Figure S11. Van't Hoff analysis of N_2 binding to [^{iBu}**1**]$^+$. Top: proposed equilibrium. Middle: Van't Hoff plot of data between 188 and 223 K. Bottom: Resulting thermodynamic parameters, based upon N_2 solubility data drawn from the literature.[51]

4. X-ray crystallographic data

4.1. $Fe(N_2)(dibpe)_2$ (^{iBu}**1·N$_2$**)

Single crystals of ^{iBu}**1·N$_2$** suitable for x-ray diffraction were obtained by cooling a pentane solution of the compound at −30 °C, forming red blocks which crystallised in the space group C2/*c*. The Fe–N–N vector lies along a crystallographic C_2 axis, such that the two dibpe ligands are related by symmetry. One of the *i*Bu substituents on the single crystallographically distinct dibpe ligand was found to be disordered across two independent orientations in a *ca*. 61:39 ratio.

CCDC deposition number 2475900 contains the crystallographic data for this structure.

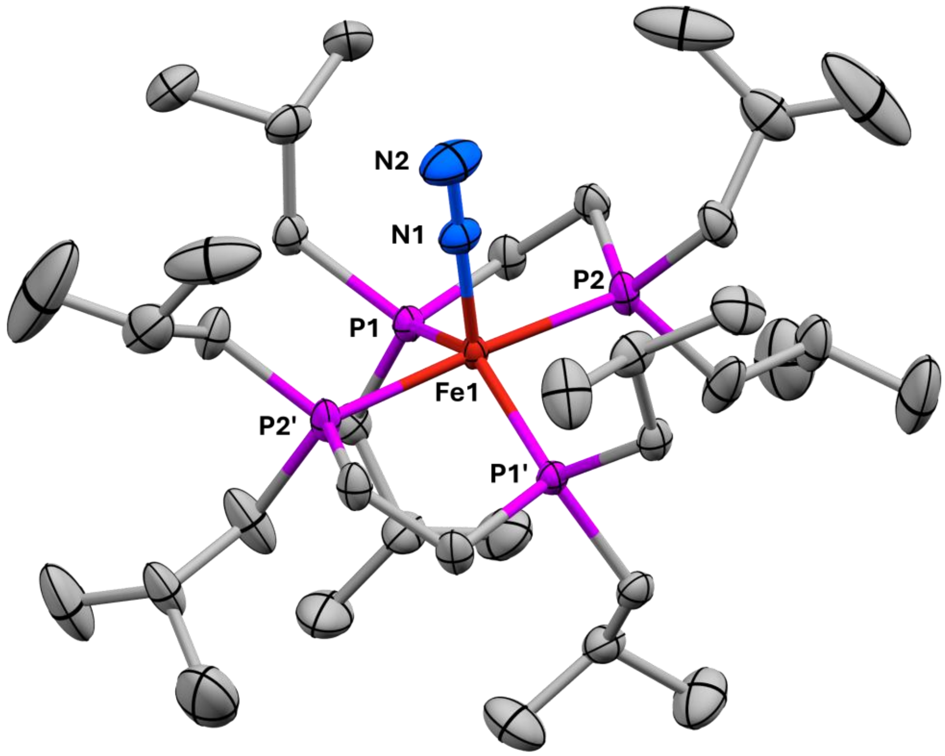

Figure S12. Solid-state molecular structure of ^{iBu}**1·N$_2$**. The ligands are related by C2 symmetry about the Fe-N_2 axis. Minor disorder positions and H atoms omitted for clarity. Atom colours are as follows: Fe, rust; N, blue; P, magenta; C, grey.

Table S4. Crystallographic data and structure refinement for ^{iBu}**1·N$_2$**.

Formula	$C_{36}H_{80}FeN_2P_4$
**Formula weight**	720.790
**Temperature/K**	172.95(10)
**Crystal system**	monoclinic
**Space group**	C2/c
**a/Å**	22.1181(7)
**b/Å**	10.2193(3)
**c/Å**	20.6326(6)
**α/°**	90
**β/°**	114.945(4)
**γ/°**	90
**Volume/Å^3**	4228.6(3)
**Z**	4
**ρ_{calc}g/cm^3**	1.132
**μ/mm^{-1}**	0.533
**F(000)**	1587.7
**Crystal size/mm^3**	0.575 × 0.434 × 0.325
**Radiation**	Mo Kα (λ = 0.71073)
**2Θ range for data collection/°**	4.06 to 54.86
**Index ranges**	-18 ≤ h ≤ 26, -12 ≤ k ≤ 5, -26 ≤ l ≤ 17
**Reflections collected**	7086
**Independent reflections**	4167 [R_{int} = 0.0329, R_{sigma} = 0.0548]
**Data/restraints/parameters**	4167/0/233
**Goodness-of-fit on F^2**	1.040
**Final R indexes [I>=2σ (I)]**	R_1 = 0.0388, wR_2 = 0.0932
**Final R indexes [all data]**	R_1 = 0.0528, wR_2 = 0.1006
**Largest diff. peak/hole / e Å^{-3}**	0.46/-0.81

4.2. [Fe(dibpe)$_2$][BAr$^F{}_4$] ([^{iBu}**1**][BAr$^F{}_4$])

Single crystals of [^{iBu}**1**][BAr$^F{}_4$] suitable for x-ray diffraction were obtained by cooling an Et_2O solution of the compound to −30 °C, forming purple blocks which crystallised in the space group P−1. The two dibpe ligands featured some disorder, with one exhibiting two positions for one of the *i*Bu substituents on P2 in a ratio of *ca.* 69:31, while the other had more significant disorder throughout the ligand. In this case, the ligand showed a pivot point near atom P4, resulting in two positions for that phosphorus atom and for two of the *i*Bu substituents. This was modelled as a single two-component system, resulting in the two positions having a ratio of *ca.* 58:42. One of the *i*Bu substituents on the other phosphorus atom of the ligand (P3) was also disordered in a ratio of 53:47. Except for two C atoms in the more disordered dibpe ligand (C35, C35′, C36, C36′) all atoms in the main fragment were refined anisotropically. Disorder was also identified in 7 of the 8 CF_3 groups in the BAr$^F{}_4{}^-$ anion, which was modelled by introducing a second minor occupancy rotational position for the F atoms. While all major occupancy F positions were refined anisotropically, this was not possible for the majority of the secondary F positions.

CCDC deposition number 2475899 contains the crystallographic data for this structure.

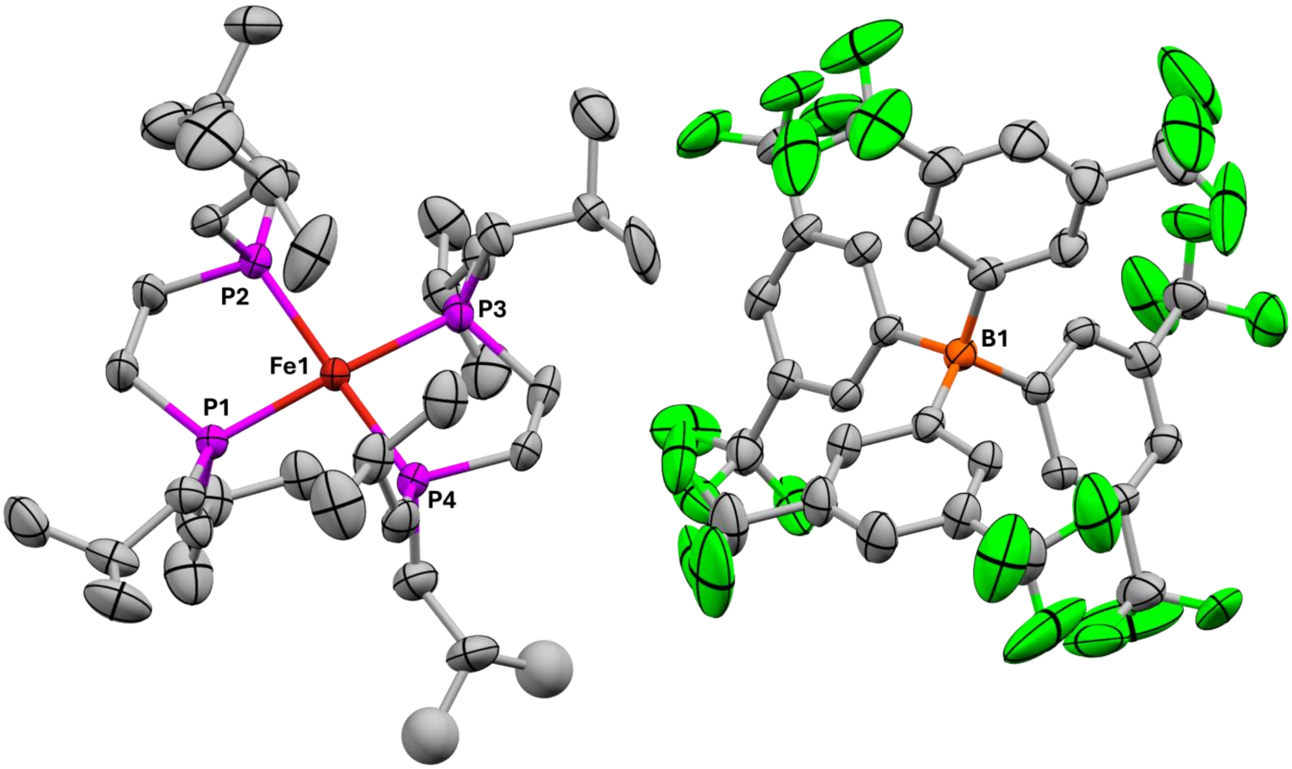

Figure S13. Solid-state molecular structure of [^{iBu}**1**][BAr$^F{}_4$]. Minor positions of disorder in dibpe ligands and CF_3 groups, and H atoms, omitted for clarity. Atom colours are as follows: Fe, rust; P, magenta; B, orange; F, bright green; C, grey.

Table S5. Crystallographic data and structure refinement for [^{iBu}**1**][BAr$^F_4$].

Formula	$C_{68}H_{92}BF_{24}FeP_4$
Formula weight	1555.95
Temperature/K	173.00(10)
Crystal system	triclinic
Space group	P-1
a/Å	14.1691(4)
b/Å	14.3171(4)
c/Å	20.1860(6)
α/°	105.754(3)
β/°	100.439(2)
γ/°	93.802(2)
Volume/Å^3	3846.9(2)
Z	2
ρ_{calc}g/cm^3	1.343
μ/mm^{-1}	0.375
F(000)	1614.0
Crystal size/mm^3	0.49 × 0.333 × 0.186
Radiation	Mo Kα (λ = 0.71073)
2Θ range for data collection/°	5.044 to 56.384
Index ranges	-18 ≤ h ≤ 17, -12 ≤ k ≤ 18, -26 ≤ l ≤ 22
Reflections collected	22230
Independent reflections	15089 [R_{int} = 0.0197, R_{sigma} = 0.0496]
Data/restraints/parameters	15089/10/1165
Goodness-of-fit on F^2	1.023
Final R indexes [I>=2σ (I)]	R_1 = 0.0507, wR_2 = 0.1116
Final R indexes [all data]	R_1 = 0.0779, wR_2 = 0.1277
Largest diff. peak/hole / e Å^{-3}	0.47/-0.34

5. References for supporting information

For refs. 1-33, refer to the main manuscript.

[34] Stoll, S., Schweiger, A., *J. Magn. Reson.* **1986**, 178(1), 42-55.
[35] Neese, F., *WIRES Comput. Molec. Sci.* **2025**, 15(1), e70019.
[36] Neese, F., *J. Chem. Phys.* **2001**, 115(24), 11080-11096.
[37] Neese, F., *J. Comp. Chem.* **2003**, 24(14), 1740-1747.
[38] Neese, F., *J. Chem. Phys.* **2005**, 122(3), Art. No. 034107.
[39] Neese, F.; Wennmohs, F.; Hansen, A.; Becker, U., *Chem. Phys.* **2009**, 356(1-3), 98-109.
[40] Helmich-Paris, B.; de Souza, B.; Neese, F.; Izs ├ík, R., *J. Chem. Phys.* **2021**, 155(10), 104109.
[41] Neese, F., *J. Comp. Chem.* **2022**, 44(3), 381.
[42] Neese, F., *J. Phys. Chem. A* **2001**, 105(17), 4290-4299.
[43] Izsak, R.; Neese, F., *J. Chem. Phys.* **2011**, 135, 144105.
[44] Izsak, R.; Hansen, A.; Neese, F., *Molec. Phys.* **2012**, 110, 2413-2417.
[45] Neese, F., *WIRES Comput. Molec. Sci.* **2012**, 2(1), 73-78.
[46] Izsak, R.; Neese, F.; Klopper, W., *J. Chem. Phys.* **2013**, 139.
[47] Neese, F., *WIRES Comput. Molec. Sci.* **2018**, 8(1), 1-6.
[48] Neese, F.; Wennmohs, F.; Becker, U.; Riplinger, C., *J. Chem. Phys.* **2020**, 152(22), 224108.
[49] Neese, F., *WIRES Comput. Molec. Sci.* **2022**, 12(1), e1606.
[50] Neese, F., *Chem. Phys. Lett.* **2000**, 325(1-3), 93-98.
[51] R. Battino, T. R. Rettich, T. Tominaga, *J. Phys. Chem. Ref. Data* **1984**, *13*, 563-600.
